# Design of synthetic bacterial communities for predictable plant phenotypes

**DOI:** 10.1371/journal.pbio.2003962

**Published:** 2018-02-20

**Authors:** Sur Herrera Paredes, Tianxiang Gao, Theresa F. Law, Omri M. Finkel, Tatiana Mucyn, Paulo José Pereira Lima Teixeira, Isaí Salas González, Meghan E. Feltcher, Matthew J. Powers, Elizabeth A. Shank, Corbin D. Jones, Vladimir Jojic, Jeffery L. Dangl, Gabriel Castrillo

**Affiliations:** 1 Department of Biology, University of North Carolina at Chapel Hill, Chapel Hill, North Carolina, United States of America; 2 Howard Hughes Medical Institute, University of North Carolina at Chapel Hill, Chapel Hill, North Carolina, United States of America; 3 Curriculum in Bioinformatics and Computational Biology, University of North Carolina at Chapel Hill, Chapel Hill, North Carolina, United States of America; 4 Department of Computer Science, University of North Carolina at Chapel Hill, Chapel Hill, North Carolina, United States of America; 5 Department of Genetics, University of North Carolina at Chapel Hill, Chapel Hill, North Carolina, United States of America; 6 Lineberger Comprehensive Cancer Center, University of North Carolina at Chapel Hill, Chapel Hill, North Carolina, United States of America; 7 Carolina Center for Genome Sciences, University of North Carolina at Chapel Hill, Chapel Hill, North Carolina, United States of America; 8 Curriculum in Genetics and Molecular Biology, University of North Carolina at Chapel Hill, Chapel Hill, North Carolina, United States of America; 9 Department of Microbiology and Immunology, University of North Carolina at Chapel Hill, Chapel Hill, North Carolina, United States of America; Max Planck Institute, Germany

## Abstract

Specific members of complex microbiota can influence host phenotypes, depending on both the abiotic environment and the presence of other microorganisms. Therefore, it is challenging to define bacterial combinations that have predictable host phenotypic outputs. We demonstrate that plant–bacterium binary-association assays inform the design of small synthetic communities with predictable phenotypes in the host. Specifically, we constructed synthetic communities that modified phosphate accumulation in the shoot and induced phosphate starvation–responsive genes in a predictable fashion. We found that bacterial colonization of the plant is not a predictor of the plant phenotypes we analyzed. Finally, we demonstrated that characterizing a subset of all possible bacterial synthetic communities is sufficient to predict the outcome of untested bacterial consortia. Our results demonstrate that it is possible to infer causal relationships between microbiota membership and host phenotypes and to use these inferences to rationally design novel communities.

## Author summary

Symbiotic microbes influence host development and health, but predicting which microbes or groups of microbes will have a helpful or harmful effect is a major challenge in microbiome research. In this article, we describe a new method to design and predict bacterial communities that alter the plant host response to phosphate starvation. The method uses plant–bacterium binary-association assays to define groups of bacteria that elicit similar effects on the host plant. By constructing partially overlapping bacterial communities, we demonstrated that it is possible to modify phosphate accumulation in the plant shoot and the induction of plant phosphate starvation genes in a controlled manner. We found that bacterial colonization of the plant root does not predict the capacity to produce this phenotype. We evaluated the predictive performance of different statistical models and identified one best able to predict the behavior of untested communities. Our work demonstrates that studying a subset of all possible bacterial communities is sufficient to anticipate the outcome of novel bacterial combinations, and we establish that it is possible to deduce causality between microbiome composition and host phenotypes in complex systems.

## Introduction

The composition of plant-associated microbial communities influences plant health and development [1][2]. This has raised interest in the use of microbes for biotechnology and agriculture [3][4]. However, it is challenging to measure the contribution of individual microbes from a complex microbiota to host health. Thus, a number of in vitro screening strategies are commonly applied to identify candidate plant-interacting microbes; however, none of the traits typically screened are correlated with a plant-beneficial outcome [5]. Another common prescreening strategy involves performing plant–bacterium binary-association assays [6], but only a few have been successfully translated into agricultural settings [7][8][9], suggesting that these assays also fail to capture critical aspects of nature’s complexity. Moreover, it is well established that microbial consortia can produce strong and unexpected effects on host health [10][11], and such emergent properties are hard to predict, hindering the rational design of microbial consortia with desired host outputs. Previous strategies to address this conceptual problem included the exhaustive study of possible communities assembled from a small number of microbiota constituents in zebrafish [12] and the analysis of randomized combinations of bacteria in mice [13]. Other approaches often begin with an exhaustive evaluation of all combinations of, for example, the nutrients nitrate and ammonium and the hormones auxin, cytokinin, and abscisic acid on plant root growth and development [14]. Although exhaustive approaches can provide a complete picture of interactions within complex systems, they are unfeasible for systems with more than a handful of variables, given the astronomical number of possible factorial combinations. Even in the rare cases in which functional microbial consortia have been assembled, most studies focus on a single community that is considered a treatment, and rarely is an effort made to dissect the contribution of its constituents. This makes it impossible to establish predictable generalizations beyond the tested communities or conditions used. An instance that dissected the components of a consortium consisted of only 2 bacterial strains [15]. These findings reinforce the necessity for reduced complexity and modular model systems to associate microbial community composition with host phenotypes.

**Fig 1:**
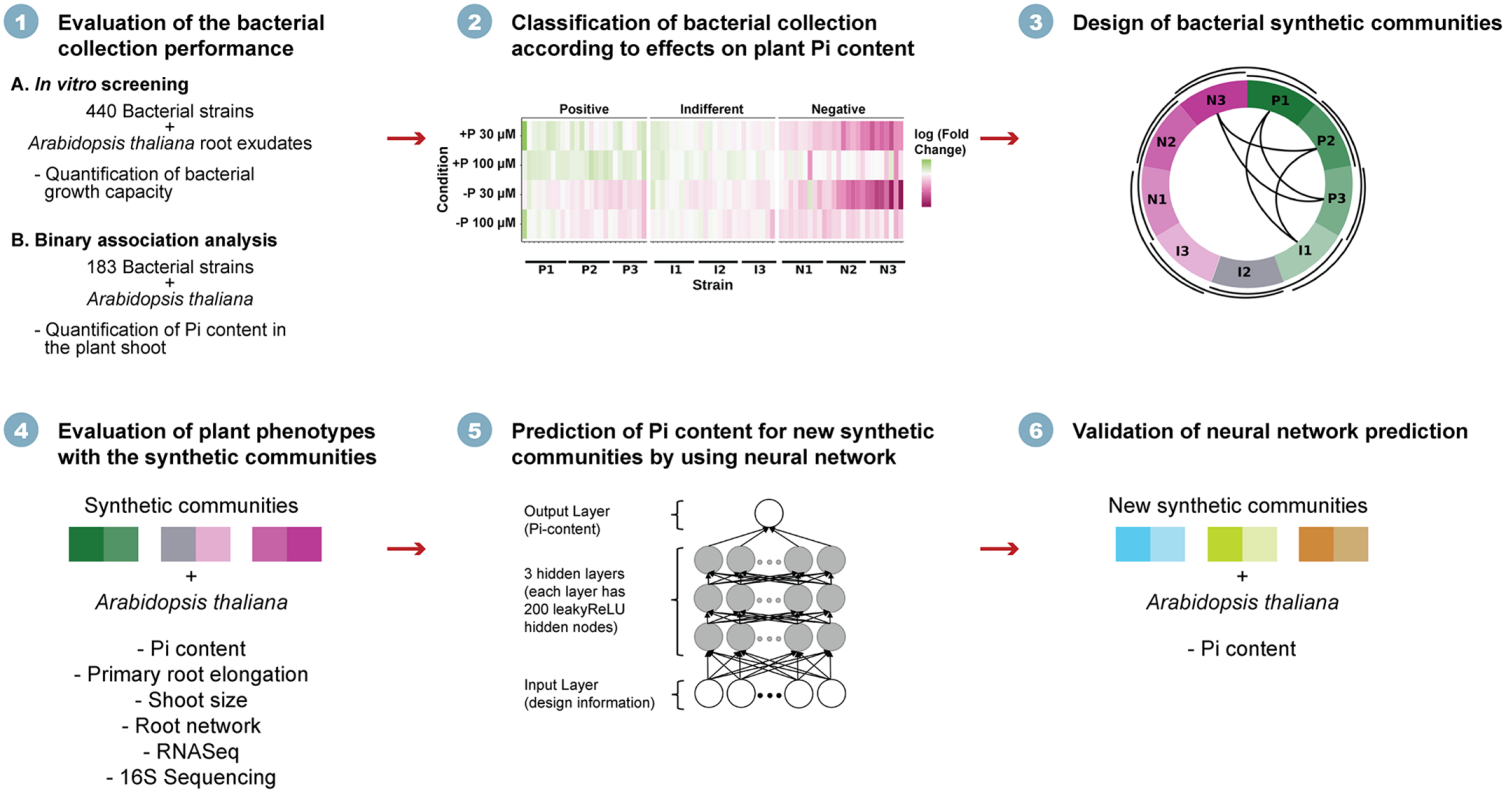
Experimental strategy to design and test small consortia of bacteria with predictable host phenotypes. I1–I3, indifferent phenotypes 1–3; leakyReLU, leaky Rectified Linear Unit; N1–N3, negative phenotypes 1–3; Pi, phosphate; P1–P3, positive phenotypes 1–3; 16S, ribosomal gene.

Our approach is summarized in Fig 1. In short, we first characterized the relationship between in vitro bacterial assays and plant–bacterium binary-association assays, and we used the latter to define functional bacterial blocks. These blocks are groups of bacteria that, by themselves, have a similar influence on a host phenotype. Then, we defined a subset of all the possible communities by constructing partially overlapping synthetic communities (SynComs) of 2 blocks each, tested the effect of these consortia on multiple plant phenotypes, and characterized the plant transcriptional response to these consortia. We evaluated the predictive performance of different statistical models on communities that the models had not seen before. We selected a neural network (NN) because it maximized predictive performance and used this model to design novel synthetic communities that maximized the change in 1 plant phenotype. Finally, we tested the model designs by constructing the novel communities it suggested and validated nearly all of the predicted host phenotypic outputs.

We focus on bacterial manipulation of the plant response to phosphate (Pi) starvation, a commonly limiting nutrient for plant growth [16]. Pi is an essential macronutrient for plants and also for microbes [17][18] and is limited in soil [19]. Microbial communities living in the proximity of the plant take up Pi from the environment using a highly efficient Pi transport system [20][21]. Therefore, the available Pi in the close vicinity of plants is subject to direct and intense competition for uptake between microbes and plants [18]. Although the response of *Arabidopsis thaliana* seedlings in axenic conditions to phosphate starvation is well characterized [22], the elucidation of the regulatory mechanisms of this response in the presence of the plant microbiome is only recently emerging [23] [24].

We systematically evaluated the performance of a large collection of root bacterial isolates using in vitro screening and binary plant–bacterium association assays as predictors for the effect of derived bacterial consortia on plant phenotypes in response to phosphate starvation. We confirm that bacterial in vitro assays have no correlation with bacterial effects on plant phenotypes. However, we found that plant–bacterium binary-association assays are informative for designing small synthetic communities. Surprisingly, the effects of bacterial consortia on host physiology were mostly additive and independent of bacterial abundances, suggesting that functional stacking within a microbial consortium can determine its effect on host phenotypic response. Finally, we successfully validated novel synthetic communities designed by an NN that led to predictable changes in plant shoot Pi content. Our results provide a useful road map from binary host–microbe assays to the design and testing of useful small consortia to predictably alter host phenotypes.

## Results

In response to Pi deficiency, plants change root exudate metabolite profiles and root architecture to explore Pi-rich soil patches [25]. This may lead to bacterial soil community shifts [26]. In order to learn how root exudate profiles change in response to Pi, we harvested root exudates from *A*. *thaliana* plants in response to 2 short and complementary nutritional transitions that mimic the dynamics of Pi stress [27] (S1A Fig; Materials and methods 1a, 1b, 1d, 1e). We demonstrated that our Pi transitions were sufficient to induce a reconfiguration of plant exudate primary metabolic profiles (S1C and S1D Fig, and S1 Table).

We next tested whether these exudates modified the in vitro growth capacity of a collection of 440 bacterial strains isolated from the roots of Brassicaceae grown in soil that is not overtly Pi deficient (nearly all from *A*. *thaliana*) ([28], S1B Fig, and Materials and methods 1c). We identified a range of bacterial growth behaviors (Materials and methods 1f, 1g) and found that the bacterial growth differences between phosphate conditions are much weaker than the differences between strains (S2 Fig). As expected, phylogeny explained most of the growth differences between strains (S2A Fig and Materials and methods 1h). Most of the bacterial growth parameters provided the same information, so we selected the area under the growth curve (optical density [OD] versus time) (AUC) as a growth marker for subsequent analyses.

Hierarchical clustering of AUC differences between in vitro conditions identified 10 groups of bacteria that represented different response patterns to exudates derived from roots grown in different Pi concentrations and media supplemented or not with Pi (S2B Fig and S2 Table). We found that root exudates could enhance or inhibit bacterial growth and that this effect could be either general or specific to one type of exudate (S2B Fig). Thus, consistent with previous findings [26], plant-derived root exudates modulated the growth of bacterial root isolates depending on the plant’s Pi starvation status.

**Fig 2:**
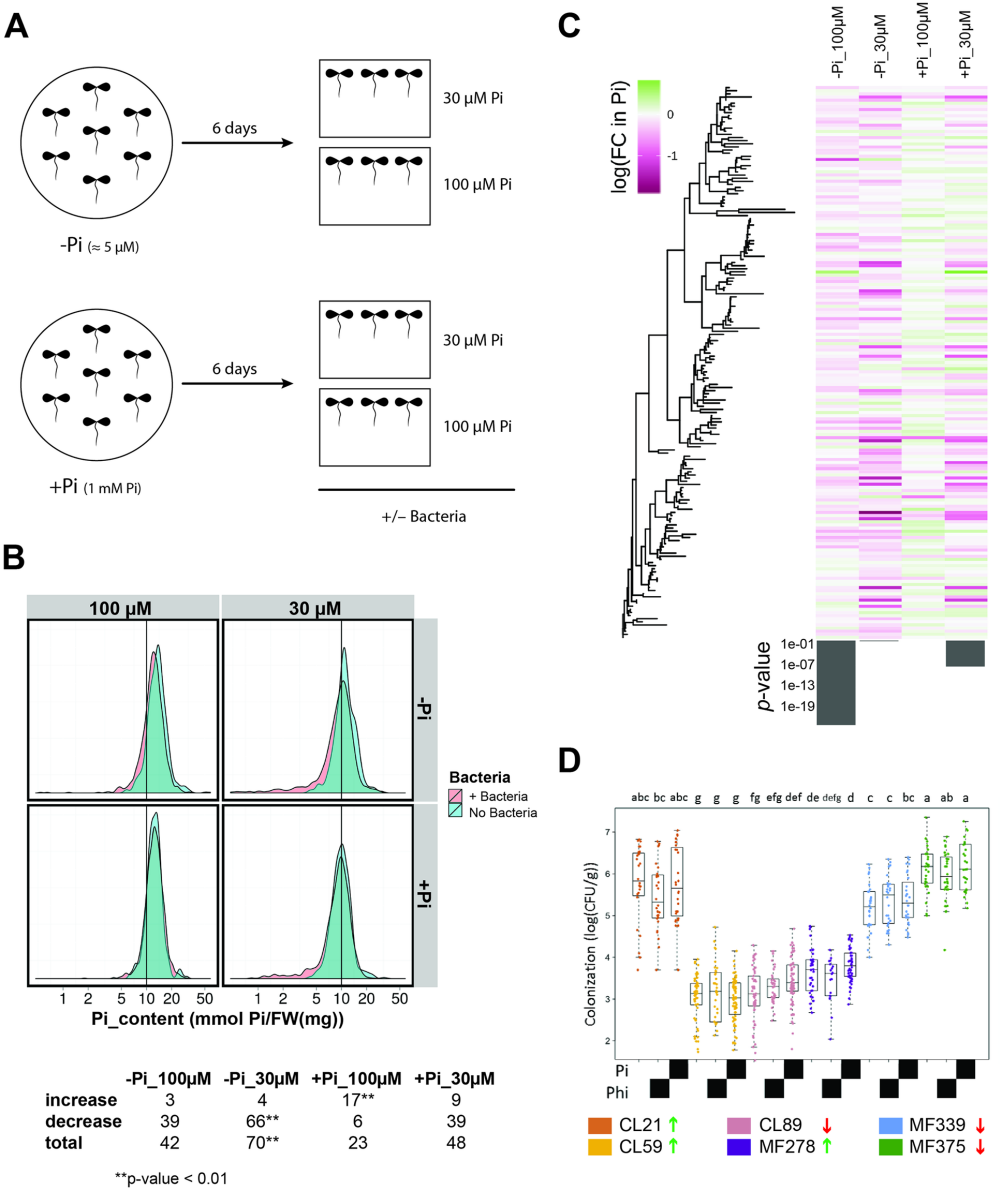
Bacteria modify the shoot Pi content in the plant. (A) Schematic representation of the pipeline used for the binary-association analysis. For binary-association experiments, plants were germinated in axenic condition on Johnson medium, 0.5% sucrose with either 1 mM Pi (+Pi), approximately 5 μM Pi [traces of Pi from the agar] (−Pi), or 1 mM phosphite (Phi; not shown) in a vertical position for 6 days. Seedlings were then transferred to 30 μM Pi and 100 μM Pi media (without sucrose), alone or with each bacterial strain, for another 7 days. *Arabidopsis thaliana* plants were grown in a growth chamber in a 16-hour light/8-hour dark regime (24°C/21°C). (B) Top: Distribution of shoot Pi content in plants cocultured with individual bacterial strains (+ Bacteria) or in axenic conditions (No Bacteria) across a 2 × 2 matrix of Pi levels used for plant germination [+Pi (1 mM Pi) and −Pi (about 5 μM Pi)] and 2 Pi concentrations (30 μM Pi and 100 μM Pi), to which seedlings were transferred concomitant with application of each bacterial strain (Materials and methods 2a). Bottom: Number of strains that significantly increase or reduce the shoot Pi accumulation compared with no bacteria, after correction for multiple testing (Materials and methods 2e). Asterisks indicate an enrichment of strains with an effect greater than expected (hypergeometric test). Bacteria (*n* = 183) and 3 replicas (10 plants each) were analyzed per strain in 2 independent experiments. See also S3 Table. (C) Heat map of log(fold-change) in shoot Pi concentration between plants inoculated with individual bacterial strains, compared with axenically grown seedlings. Treatments are as in (B) and bacteria are sorted according to their phylogeny, as indicated by the tree on the left. Bottom bar plot shows the *p*-value from Pagel’s *λ* test for phylogenetic signal. Only 177/183 strains that were both tested in the plant–bacterium interaction assays and had a high-quality full-length 16S sequence are included. (D) Colonization capacity of 6 bacterial strains selected according to their performance in binary-association assays: 3 strains increased (green arrows) and another 3 decreased (red arrows) the shoot Pi content (see S3E Fig). CL and MF refer to the natural soils from which the strains were isolated. For this experiment, we used plants germinated with (black block) or without (no block) Pi or in the presence of phosphite (black block). Plant tissue was crushed; serially diluted, plated, and c.f.u’s per gram of original material were determined. Data points are colored by bacterial strain. Letters at the top of each panel denote statistical significance of Tukey’s post hoc analysis of a linear model. Numerical values that underlie the data displayed in the panel are in https://github.com/surh/wheelP. c.f.u, colony-forming unit; FW, fresh weight; Phi, phosphite; Pi, phosphate; 16S, ribosomal gene.

We selected a subset (*n* = 183) of the strains from the in vitro assays for determining whether they exerted a functional role on the plant under different phosphate conditions. We selected bacterial isolates that belonged to all of the different response patterns (S2B Fig) and that were most responsive to both Pi levels and the presence of exudates (Materials and methods 1g, S2 Table). We measured the change in plant shoot Pi content, a direct marker of phosphate starvation responsiveness [22], in response to the presence of each of 183 individual strains, when compared to axenically grown plants. We evaluated shoot Pi content under 4 Pi conditions that represented a 2 × 2 design matrix of 2 Pi levels used for plant germination (full; 1 mM; and depleted, about 5 μM Pi) and 2 Pi concentrations (30 μM Pi and 100 μM Pi) to which seedlings were switched, concomitant with the application of each bacterial strain (Fig 2A and Materials and methods 2). The use of 2 germination conditions in the experimental design allowed us to evaluate the effect of the activation of the phosphate starvation response and the shoot Pi content on the plant–bacterium interaction under different Pi concentrations.

On average, bacteria had a slightly negative effect on plant shoot Pi content, visualized as a small tail in the bacterial treatment graphs (pink) in Fig 2B. This effect was stronger when the environmental Pi concentration was lower (Fig 2B and 2C and S3 Table). These findings are consistent with our previous results that a bacterial synthetic community drives a context-dependent competition with the plant for Pi [23]. Overall, we found that more strains had a negative than a positive effect on shoot Pi content (Fig 2B, S3 Table, and Materials and methods 2e). Specifically, there were significantly more strains that had a stronger negative effect on plant shoot Pi content in the most limiting Pi conditions (germination in Pi depleted, followed by transfer to 30 μM Pi) (Fig 2B and S3 Table), in which the phosphate starvation response should be active. Conversely, the least Pi-deprived condition (germination in full Pi, followed by transfer to 100 μM Pi) exhibited a significant enrichment of strains that positively affected shoot Pi content (Fig 2B and S3 Table). These results are consistent with bacterial effects on Pi content in the shoot being modulated by the nutritional status of the plant. Importantly, germination conditions did not alter bacterial colonization (Fig 2D), and the effect of individual strains on plant shoot Pi content was independent of the ability of root-inoculated bacteria to colonize the shoot and independent of bacterial titers in different plant organs (Fig 2D; S3A Fig and Materials and methods 2c).

We detected a weak phylogenetic signal in the ability of bacterial strains to modulate plant Pi content that was significant in only 2 of the 4 conditions (Fig 2C and Materials and methods 2f). Accordingly, we found no correlation between the effect of individual bacterial isolates on shoot Pi content and their in vitro growth phenotype in response to switched Pi levels and root exudates (S4 Fig). Overall, our survey of plant–bacteria binary associations and the resulting distribution of bacterial effects on shoot Pi content argue that the majority of plant–bacteria interactions are competitive, at least in the context of phosphate starvation response.

We recently demonstrated that the *A*. *thaliana* phosphate starvation response is largely antagonistic to immune system function [23]. We therefore asked whether activation of the plant phosphate starvation response could modulate the outcome of binary bacteria–plant interactions. We analyzed shoot Pi content in plants pretreated with phosphite (Phi) (KH_2_PO_3_) and then transferred to either 30 μM Pi or 100 μM Pi in the presence of each of 30 selected bacterial strains that either reduced, increased, or had no effect on the shoot Pi content (10 strains per class; S3E Fig). Phi is a nonmetabolizable analog of Pi and its accumulation delays the phosphate starvation response, resulting in low accumulation of Pi in the shoot [29](S3B and S3C Fig). We found that germinating plants on Phi (low shoot Pi, phosphate starvation response off) dramatically reduced the number of bacterial isolates that diminished shoot Pi content, compared to germination on low Pi (low shoot Pi, phosphate starvation response on) (S3D and S3E Fig). Additionally, we observed that under Phi pretreatment, none of the strains significantly increased shoot Pi content compared to germination on high Pi (high shoot Pi, phosphate starvation response off) (S3D and S3E Fig). Importantly, Phi treatment did not alter bacterial colonization (Fig 2D, S3A Fig and Materials and methods 2c). These findings indicate that activation of the plant phosphate starvation response results in different modes of bacterial interactions with the plant that are independent of shoot phosphate content. These results indicate that an active phosphate starvation response can modulate the outcome of both positive and negative interactions with bacteria, likely mediated via coregulation of the plant immune system. An analogous mechanism has been described for the interaction between *A*. *thaliana* and a beneficial fungus [24].

**Fig 3:**
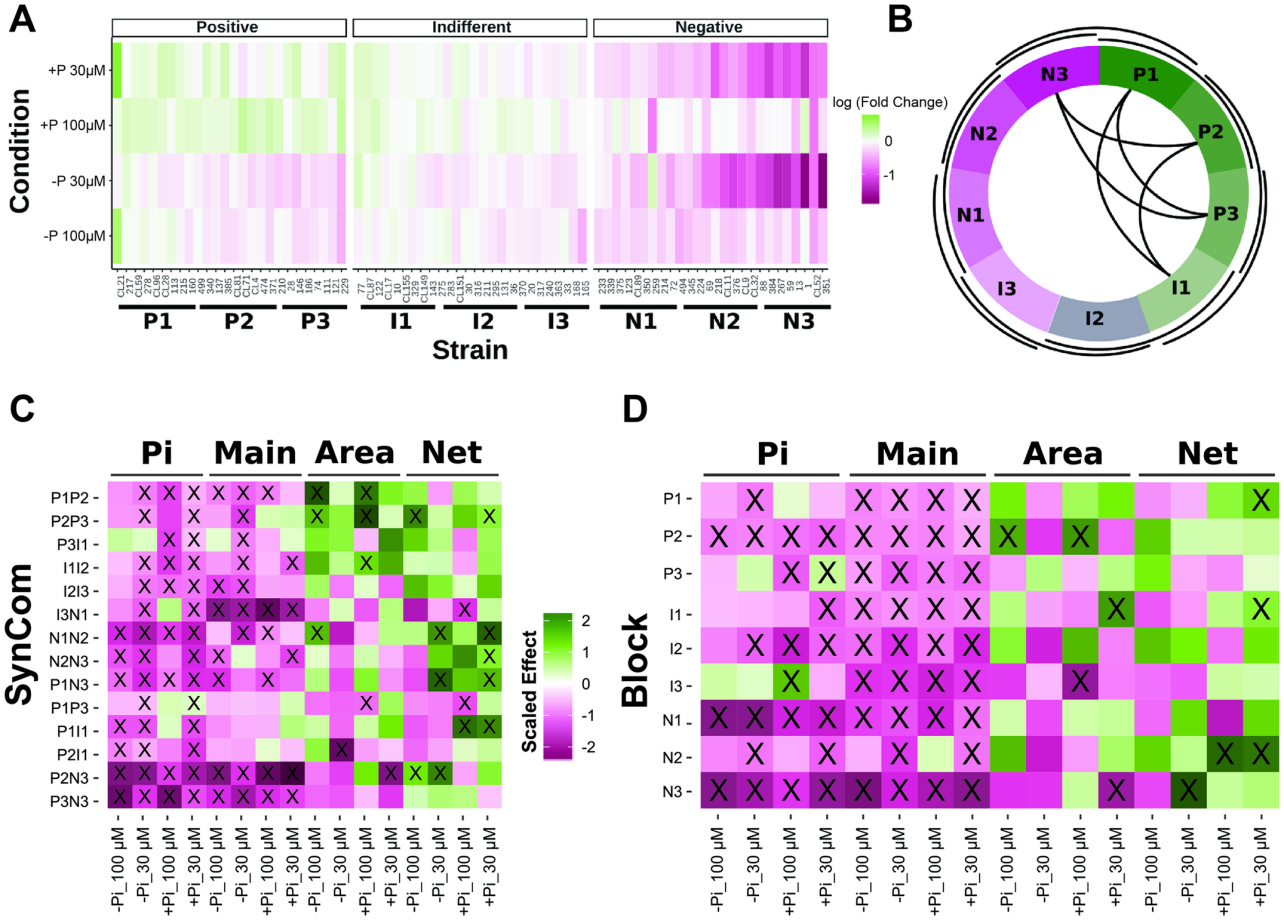
Synthetic communities alter plant phenotypes according to the strain makeup of the blocks from which they were composed. (A) Heat map showing strains (*n* = 78) tested in binary association and that were selected because they cause positive (P), negative (N), or indifferent (I) effects on shoot Pi content in the growth conditions defined in Fig 2A. Strains are sorted within each group according to their mean effect on shoot Pi accumulation. Color scale shows log(fold-change) of shoot Pi content with respect to axenically grown plants. Bars and labels at the bottom show the 9 bacterial blocks used for the design of synthetic communities. Log(fold-change) is calculated from 6 pools of 10 plants in 2 independent experiments. See also S3 and S4 Tables. (B) Schematic representation of the synthetic communities designed using pairs of blocks. Sections in the circle are the 9 bacterial blocks from (A); black curved segments represent synthetic communities. Outer curved segments and curves inside the circle represent synthetic communities made of adjacent and nonadjacent bacterial blocks, respectively. (C) Heat map shows the scaled effect of each synthetic community on 4 plant phenotypes: Pi content (Pi), primary root elongation (Main), shoot area (Area) and total root network (Net) across the 4 growth conditions defined in Fig 2A. (D) Similar to (C) for individual bacterial functional blocks. In both (C) and (D), the values correspond to the scaled coefficients from a linear model. The values have been scaled through dividing by the standard deviation of all coefficients for the same phenotype and condition (each column in the plots). In all cases, 0 (white) represents no change with respect to axenically grown plants. The method to estimate the block and synthetic community effects are described in Materials and methods section 3j, and statistical significance (*p*-value < 0.05) is indicated with an “X” inside each tile, while the results of testing for significance for changes in Pi content are presented in S5 Table. Area, shoot area; Pi, phosphate; SynCom; synthetic community.

We sought to establish whether the results from binary associations are indicative of bacterial effects when a more complex bacterial community is present. We used a microcosm reconstitution approach, in which we inoculated plants with defined complex bacterial synthetic communities (Materials and methods 3a). A subset of 78 strains analyzed in the binary-association experiments was grouped into 3 functional groups consisting of positive (P1-P3), indifferent (I1-I3), and negative (N1-N3) bacteria, depending on their effect on shoot Pi accumulation. For the positive and negative groups, we focused on strains that had a statistically significant effect on plant Pi accumulation, after correcting for multiple testing (Materials and methods 3i). Each functional group was further divided into another 3 blocks of 8–9 bacterial strains, according to the magnitude of their individual effects (Fig 3A, S3 and S4 Tables, and Materials and methods 3i). We then combined pairs of these blocks to define 14 partially overlapping bacterial synthetic communities (Fig 3B). This scheme was designed to maximize the probability of observing extreme plant phenotypes by stacking functionally similar blocks and to gain information from combining the most extreme phenotypic blocks defined in the binary-association assays.

We evaluated shoot Pi content, primary root elongation, shoot size, and total root network in *A*. *thaliana* plants grown in association with the 14 bacterial synthetic communities in the same growth conditions used for the binary-association analysis (Fig 2A and Materials and methods 3b). We found that synthetic communities, like individual bacterial strains, were more likely to reduce plant shoot Pi content, and that synthetic communities made of negative blocks led to lower shoot Pi accumulation than those composed of positive blocks (Fig 3C and 3D and S5 Fig). For example, in general, the estimated negative effect on shoot Pi accumulation for negative blocks is significantly larger than for positive or indifferent blocks (Fig 3C and 3D, S5a Table, and Materials and methods 3j). At the synthetic community level, the effect of the negative strains was clearly dominant; only 2 communities containing negative blocks (I3N1 and N2N3) showed a nonsignificant reduction in shoot Pi content and this in only 1 of the tested conditions. Importantly, the only significantly positive effect with respect to no bacteria involved 2 positive blocks and was weak (P1P3) (Fig 3C, S5b Table, and Materials and methods 3j). We also observed that the majority of the cases in which a synthetic community did not significantly reduce the shoot Pi accumulation occurred under the less Pi-restricted condition (100 μM) (Fig 3C, S5b Table, and Materials and methods 3j), consistent with the results from the individual strains (Fig 2B). This trend was generally consistent for the other plant phenotypes analyzed (Fig 3C and 3D and S5 Fig). Overall, the reduction in shoot Pi content associated with negative blocks correlated with less shoot area, shorter primary roots, and bigger root networks (top and bottom rows in Fig 3C and 3D, and S5 Fig), morphological changes that match the canonical phosphate starvation response in axenic conditions [22][30]. In contrast, positive bacterial blocks caused less intense plant phosphate starvation response phenotypes. These effects were more obvious in plants grown at low environmental Pi concentration (Fig 3C and 3D and S5 Fig). Thus, the binary-association assays were generally informative with regard to the behavior of bacteria in more complex biotic backgrounds.

Interestingly, we observed that a number of synthetic communities, for example P2P3 and P1P2, led to increased shoot area compared to axenically grown plants, despite exhibiting reduced shoot Pi content (Fig 3C and S5 Fig). In contrast, plants treated with P1P3 in +Pi_100 μM Pi condition, had shoot Pi content similar to Pi-sufficient plants but unexpectedly exhibited a reduced shoot area (Fig 3C and S5 Fig). Thus, bacterial consortia can decouple shoot Pi-content accumulation from the growth inhibition responses typically associated with the canonical phosphate starvation response [22][30][31].

**Fig 4:**
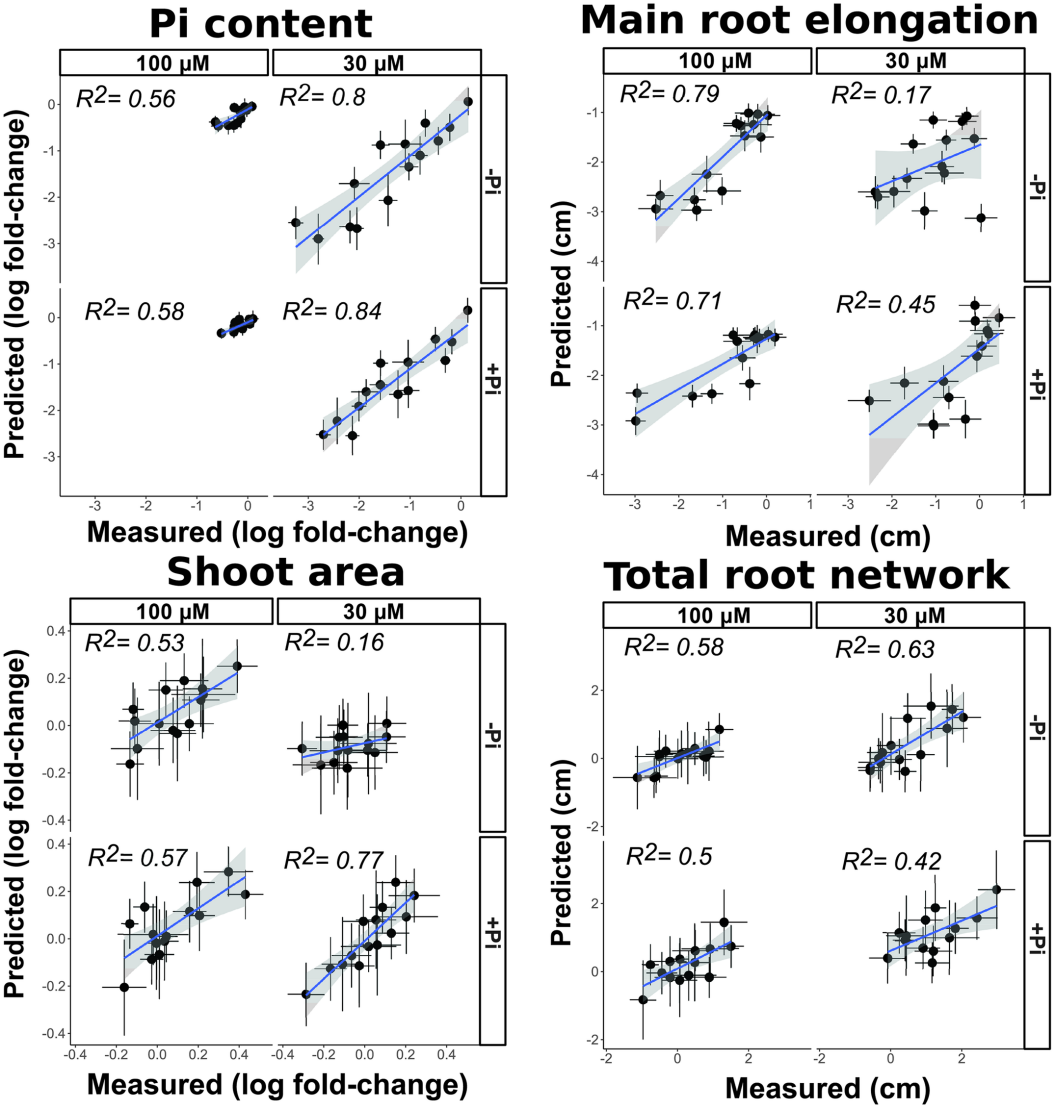
Synthetic communities additively modulate plant phenotypes. Additive contributions of bacterial blocks explain synthetic community effects on all plant phenotypes. Comparisons between measured changes (x-axis) in plant phenotypes caused by synthetic communities, with respect to axenically grown plants, and expected changes (y-axis) from purely additive effects of each block, while ignoring bacterial relative abundances. In each plot, the 4 panels represent the 4 media conditions tested, with germination conditions as rows and Pi treatment as columns. Each point represents a synthetic community (*n* = 14); the x-axis corresponds to the color scale in Fig 3C, and the y-axis shows the result from adding the individual main effects estimated for each block (Fig 3D). The standard error from both the measured and estimated change is shown for each point. The blue line represents the least squares regression on the points from each panel, and the grey shade indicates the 95% confidence interval on the regression line. *R*^2^ is shown on each panel. For all axes, 0 represents no change with respect to axenically grown plants. The values for Pi content and shoot area are indicated as log(fold-change) with respect to axenically grown plants. The values for primary root elongation and total root network represent the difference with respect to axenically grown plants. Additivity is evidenced by agreement between predicted and measured phenotypic changes. Numerical values that underlie the data displayed in the panels are in https://github.com/surh/wheelP. Pi, phosphate; *R*^2^, coefficient of determination.

We estimated the common (additive) effects of each block of strains across different bacterial backgrounds (e.g., in different synthetic communities) (Materials and methods 3j). Surprisingly, we found that additive contributions of the bacterial functional blocks are sufficient to explain most of the plant phenotypic variation observed (Fig 4). We found that synthetic community membership (i.e., ignoring bacterial relative abundances) typically explained more than 50% of the plant phenotypic variance (Fig 4). This indicates that intrablock bacterial interactions contribute at least as much as interblock interactions to the plant phenotypes tested. Furthermore, the effect of bacterial blocks on the phenotypes analyzed is generally consistent across different synthetic communities, despite each strain’s relative abundance being dependent on the microbial context (S6 and S7 Figs).

We found that the bacterial abundances in either agar substrate or in the root endophytic compartment were poorly correlated with plant phenotypes (Materials and methods 3e, 3k). Despite the consistent taxonomic profiles of the inoculum, we observed that bacterial communities of agar and root samples were dominated by variable bacterial taxa, depending on the specific combination of bacterial blocks present (S6A and S6B Fig). This suggests that bacteria–bacteria interactions are important in shaping the final community. Furthermore, we found clear taxonomic differences between root and agar samples. Most notably, *Streptomyces* strains (order Actinomycetales) were particularly good root colonizers despite their limited success on agar, while Pseudomonadales strains were relatively more successful in agar than in root samples (S6A and S6B Fig). These results recapitulate previous findings in natural soils, indicating that Actinobacteria are enriched in *A*. *thaliana* roots [32][33]. Phosphate concentrations in the media had only a minimal effect on the final community composition (S6B Fig).

We then quantified the information gained by incorporating relative abundance data (S6 Fig) into our additive model (Materials and methods 3k). Surprisingly, in all cases (16/16) the plant phenotypic variance explained by microbiota composition decreased when we incorporated relative abundance (S7 Fig). While in some cases, the differences might not be statistically significant, together, this result demonstrates significantly better performance by the model that ignores relative abundance (*p*-value = 0.000481; 2-tailed Wilcoxon signed-rank test). Our results indicate that bacterial blocks disproportionately modulate shoot Pi content with respect to their strain abundances, an observation analogous to that seen in bacteria modulating zebrafish immune responses [12].

**Fig 5:**
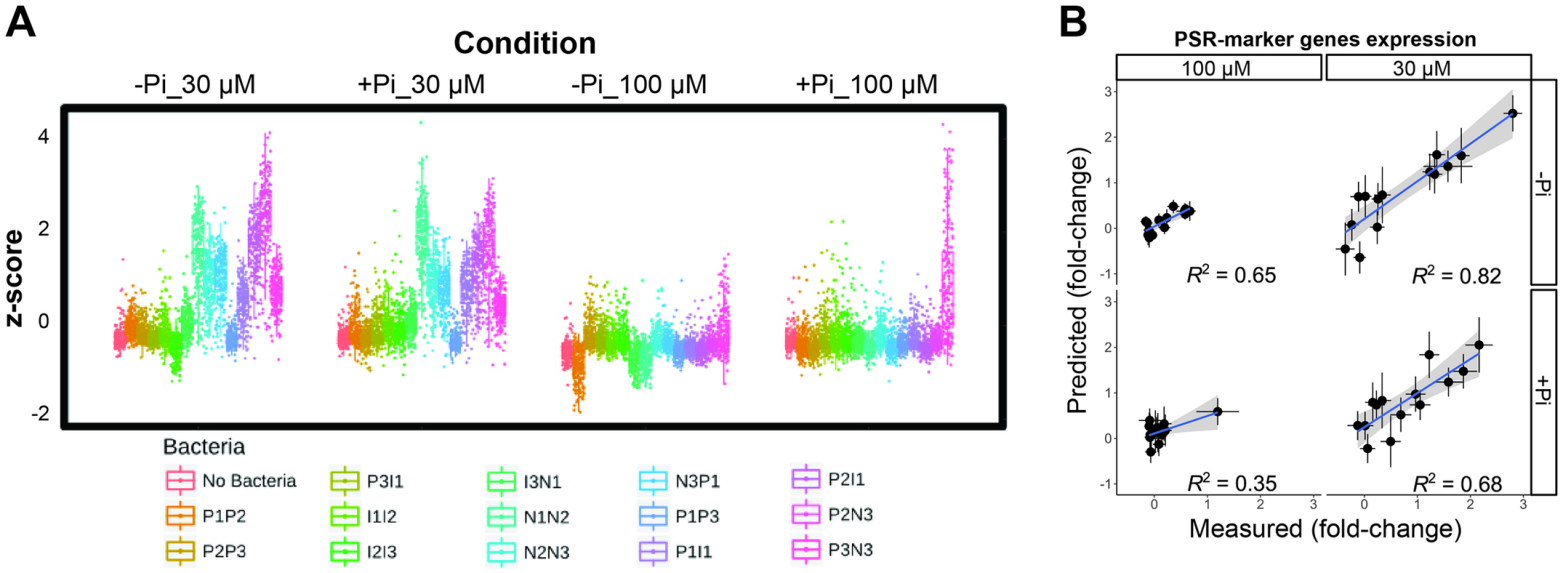
Synthetic communities additively induce the expression of phosphate starvation response marker genes in the plant. (A) Average expression of a core of 193 phosphate starvation response marker genes in plants cocultured with synthetic communities in 4 growth conditions defined in Fig 2A. (B) Additive contributions of bacterial blocks explain synthetic community molecular phenotypes. Comparisons between the phosphate starvation response marker gene expression in (A) caused by synthetic communities, with respect to axenically grown plants and expected changes (y-axis) from purely additive effects of each block. In the plot, the 4 panels represent the 4 media conditions tested, with germination conditions as rows and Pi treatment as columns. Each point represents a synthetic community (*n* = 14), and the standard error for the measured and predicted change is shown. The blue line represents the least squares regression on the points from each panel, and the grey shade indicates the 95% confidence interval on the regression line. *R*^2^ is shown for each condition. For both axes, 0 represents no change with respect to axenically grown plants. Numerical values that underlie the data displayed in the panel are in https://github.com/surh/wheelP. Pi, phosphate; PSR, phosphate starvation response; *R*^2^, coefficient of determination.

The synthetic communities differentially modulated plant phenotypes related to phosphate starvation response. Therefore, we examined the transcriptomes of plants growing with different synthetic communities. We first explored the expression of a literature-based core set of 193 phosphate starvation response transcriptional markers [23]. Plants did not exhibit induction of phosphate starvation response markers in axenic conditions, even when Pi was low (Fig 5A) [23]. However, some synthetic communities induced the canonical transcriptional response to Pi starvation in plants grown on 30 μM Pi (Fig 5A). Plants that showed transcriptional activation of the phosphate starvation response displayed lower shoot Pi accumulation. However, we also observed that some synthetic community treatments lead to low shoot Pi content and no activation of the transcriptional phosphate starvation response (S8 Fig). The effect of synthetic communities was in general dependent on the presence of negative bacterial strain blocks (Fig 5A). In contrast, synthetic communities consisting of only positive blocks of bacteria did not induce the phosphate starvation response transcriptional signature in any condition analyzed (Fig 5A). No induction of the phosphate starvation response genes was observed when the Pi stress was released (following transfer to 100 μM Pi) except for the bacterial combination P3N3, which exhibited induction on 100 μM Pi (Fig 5A). In accordance with the shoot Pi content data (Figs 3C, 3D and 4), we found that additive effects of bacterial blocks could explain the level of transcriptional induction (Fig 5B). The specificity in the bacterial modulation of plant phenotypes suggests that the changes observed in the plant in response to the synthetic communities are linked to bacterial block activities.

We next explored the overall plant genome-wide transcriptional response to bacteria consortia, Pi conditions, or both. Our design allowed us to test both the response to synthetic communities and to individual bacterial blocks between and within conditions (Materials and methods 3h). As anticipated, plants growing with bacterial synthetic communities on low Pi generally induced phosphate starvation responsive genes and modified the expression of immune system–related genes (S9 Fig, S6 and S7 Tables) [23]. Overall, there was not a common response to bacterial presence, with only 45 and 35 genes being significantly up- or down-regulated by more than half of the bacterial blocks, respectively (S6 Table). The number of genes differentially expressed in response to different bacterial blocks did not correspond with the strain composition of the blocks; blocks P2, N3, and I1 altered the expression of the most genes, and blocks I3, N1, and P3 influenced the least (S6 Table). In particular, block I3 only altered the expression of 17 genes, despite being detected in plant roots and surrounding agar (S6 Fig). At the functional level, most of the bacterial blocks induced the expression of the plant defense response, specifically up-regulating genes for salicylic acid biosynthesis (S10 Fig), consistent with overall Bacteria versus No Bacteria comparisons (S9C Fig).

**Fig 6:**
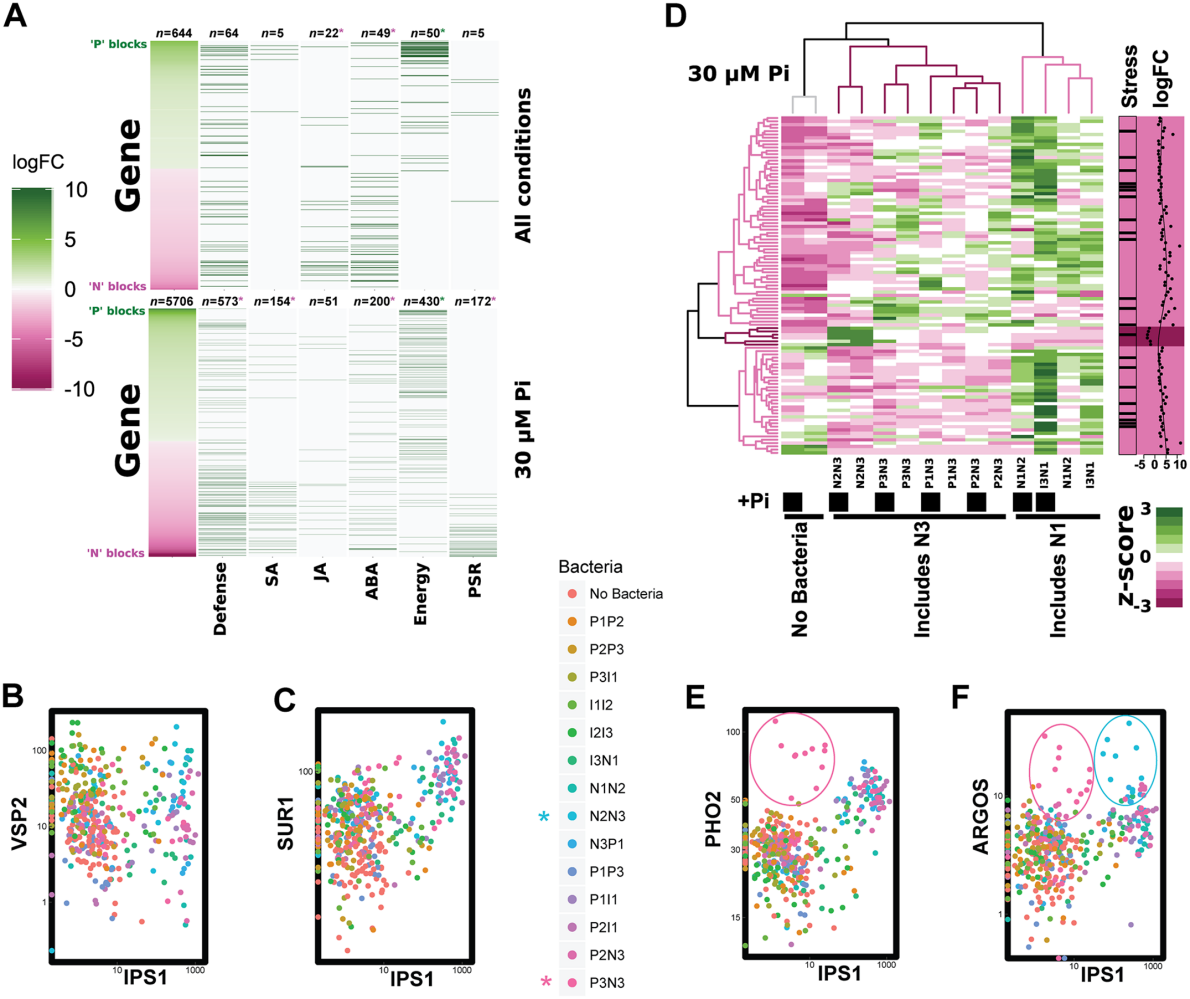
Synthetic communities modify plant transcriptional profiles. (A) Comparison of differentially expressed genes between all the positive (green) and all the negative blocks (magenta), in all conditions (top) or at 30 μM Pi (bottom). The first column shows all differentially expressed genes sorted by their log(fold-change), while the following columns indicate different functional annotations. Numbers at the top of each column show how many genes are marked and colored asterisks indicate a significant enrichment of the function among genes more expressed by positive (green asterisk) or negative (magenta asterisk) blocks. Panels (B) and (C) compare the expression of *IPS1*, a gene activated by low Pi, with (B) the JA response marker *VSP2* and (C) the glucosinolate biosynthesis marker *SUR1*. Expression values are RPKM on a log_10_ scale. (D) Comparison of 103 differentially expressed genes between 2 negative bacterial blocks (N1, N3) under low Pi (30 μM) condition. Rows represent genes and columns specific synthetic communities under different conditions. Color in the heat map shows the average expression of the corresponding genes across 2 independent experiments (3 replicates per experiment). Hierarchical clustering dendrograms are shown for both genes and conditions. Color in the dendrogram indicates the block that is included in the corresponding condition (column) or that up-regulates the corresponding gene (columns). Darker magenta color corresponds to block N3, and lighter magenta color corresponds to block N1, as in Fig 3B. Genes involved in stress response (Stress) are indicated on the right, and the logFC in expression between blocks N1 and N3 is also indicated, with positive values indicating a higher expression in the presence of block N1. Panels (E) and (F) compare the expression of *IPS1* with (E), a phosphate starvation response–induced ubiquitin-conjugating E2 enzyme, *PHO2* (F), and an auxin-regulated gene, *ARGOS*. Expression values are RPKM in a log_10_ scale. Ellipses highlight samples from plants inoculated with synthetic communities P3N3 (pink asterisk) and N2N3 (blue asterisk). For panels (B), (C), (E), and (F), points on the axes represent samples in which the expression of the corresponding gene was not detected. ABA, abscisic acid; *ARGOS*, AUXIN-REGULATED GENE INVOLVED IN ORGAN SIZE; *IPS1*, INDUCED BY PHOSPHATE STARVATION1; JA, jasmonic acid; logFC, log(fold-change); *PHO2*, PHOSPHATE2; Pi, phosphate; PSR, Pi starvation response; RPKM, reads per kilobase per million; SA, salicylic acid; Stress, stress response; *SUR1*, SUPERROOT1; *VSP2*, VEGETATIVE STORAGE PROTEIN2.

We also investigated differences between the genes induced by different bacterial blocks. Comparison of genes differentially expressed between positive and negative blocks across all conditions showed that positive blocks had higher expression of genes involved in energy production, while negative blocks specifically induced abiotic stress–responsive gene sets, specifically abscisic acid–related genes (Fig 6A, S6 and S7 Tables). Negative blocks of bacteria also increased the expression of a specific sector of the jasmonic acid response involved in glucosinolate biosynthesis (Fig 6A–6C, S6 and S7 Tables). The glucosinolate pathway modulates the interaction of *A*. *thaliana* with a beneficial fungus at low Pi [24], and its expression is regulated by the master regulator of phosphate stress response, PHR1 (PHOSPHATE STARVATION RESPONSE1) [23]. When the environmental Pi was low (30 μM Pi), we observed many more differentially expressed genes between positive and negative blocks (Fig 6A), with negative blocks driving higher expression of genes of both the phosphate starvation and defense response.

We then focused our analysis on the Pi-limiting conditions (30 μM). In this condition, synthetic communities containing negative blocks showed a strong induction of the phosphate starvation response (Fig 5A). We asked whether the different negative blocks (N1, N2, and N3) differed in their effects. There were almost no expression differences between the 2 most negative blocks (N2 and N3), but we identified 103 genes differentially regulated by bacterial blocks N1 and N3. These genes were mostly stress-related genes, including general abiotic stress and defense response, the expression of which was comparatively reduced in the phenotypically more negative block N3 (Fig 6D, S6 and S7 Tables). This result indicates that under phosphate starvation, all negative blocks activate a similar set of phosphate starvation response genes but differentially suppress other stress responses.

We found that some genes were induced in response to specific block combinations. For example, we found that PHOSPHATE2 (*PHO2*), a ubiquitin-conjugating E2 enzyme in *A*. *thaliana* required for the degradation of Pi transporters at high Pi [34], is highly expressed only in plants exposed to the synthetic community P3N3 in all Pi conditions analyzed (Fig 6E). This finding may explain the strong transcriptional response to Pi starvation caused by this synthetic community (Fig 5A). The auxin-regulated gene AUXIN-REGULATED GENE INVOLVED IN ORGAN SIZE (ARGOS) [35] showed a weak positive correlation with the induction of the phosphate starvation response, and it was induced in plants grown with the synthetic communities P3N3 and N2N3 (Fig 6F). ARGOS controls organ size in *A*. *thaliana* and its transgenic expression results in enlarged aerial organs [35]. This could serve to counterbalance the negative effect on shoot size that low Pi typically causes.

**Fig 7:**
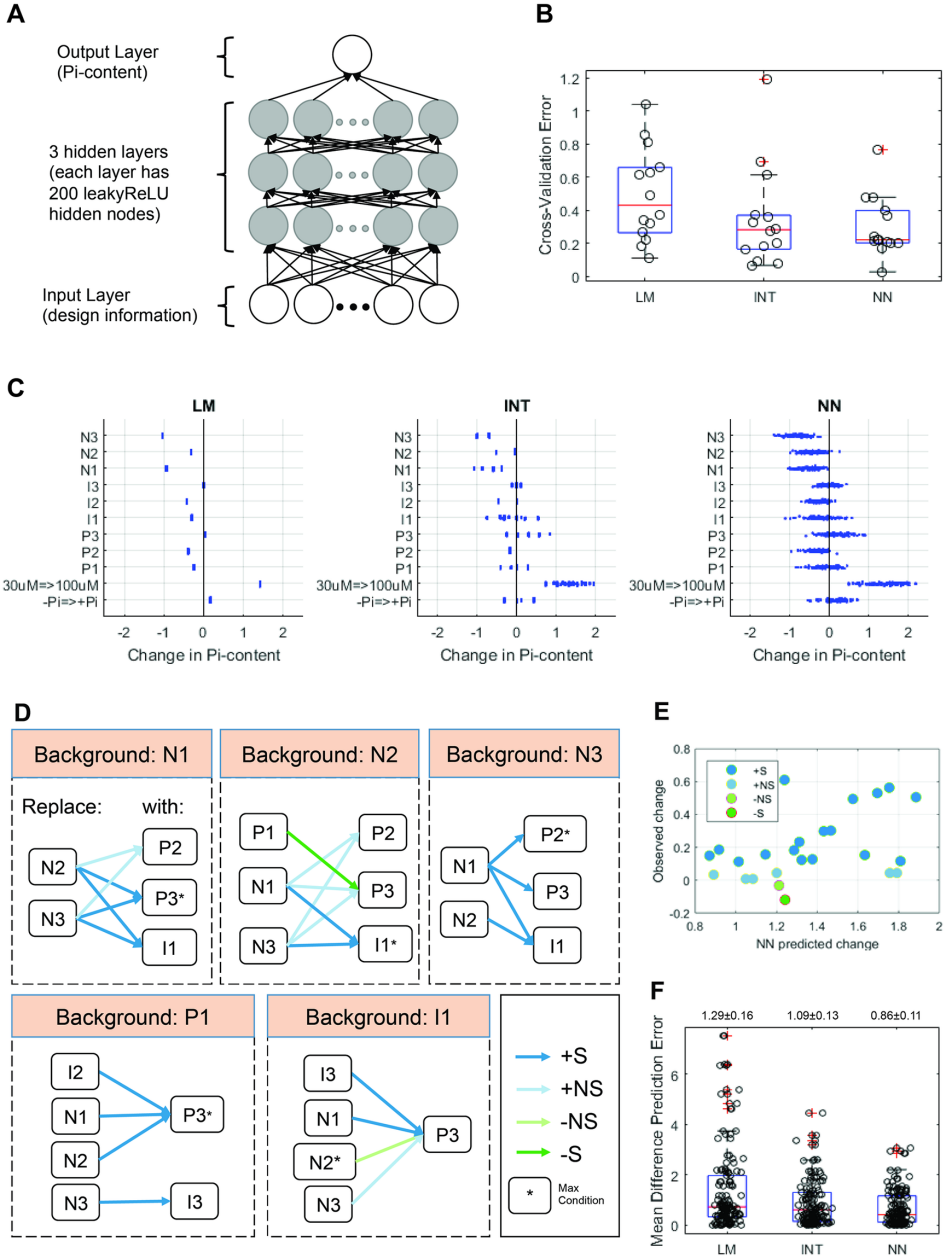
The effect of novel synthetic communities on plant shoot Pi content can be predicted by an NN. (**A**) Schematic representation of the NN defined and applied for predictions. Nodes are neurons, and arrows are weights that are estimated from the data. (**B**) Cross-validation error from the 3 types of models tested for their ability to predict shoot Pi content. Each model is trained on all but one synthetic community and evaluated on that held-out synthetic community. Each dot in the plot represents the mean Pi content prediction error on the held-out synthetic community. (**C**) Sensitivity of Pi accumulation with respect to each biological variable for each type of model. Each dot represents the change of shoot Pi content under a specific combination of input conditions (Materials and methods 4f). (**D**) The 25 most significant block replacements with a positive effect on the shoot Pi concentration predicted by the NN. These block replacements involved 20 different synthetic communities. Each box represents selected replacements in a particular constant background noted at the top. Each arrow represents a replacement of the bacterial block on the left with the block on the right. Asterisks indicate the blocks that lead to maximal plant Pi accumulation in the validation experiment. (**E**) The shoot Pi accumulation change predicted by the NN (x-axis) and the change observed experimentally are significantly correlated (Spearman’s correlation coefficient 0.42, *p*-value = 0.0375). For (**D**–**E**), color represents the validation experimental result: significant increase (dark blue), nonsignificant increase (light blue), nonsignificant decrease (light green), and significant decrease (dark green). (**F**) Prediction error on all tested block replacements for the LM, INT, and NN. The mean prediction error values ± standard errors are indicated above each box. The validation prediction error on NN is significantly smaller than LM (*p*-value = 5.25 × 10^−10^) and INT (*p*-value = 4.65 × 10^−7^). Numerical values that underlie the data displayed in the panels are in https://github.com/surh/wheelP. −NS, nonsignificant decrease; −S, significant decrease; +NS, nonsignificant increase; +S, significant increase; INT, linear model with interaction; leakyReLU, leaky Rectified Linear Unit; LM, linear model; NN, neural network; Pi, phosphate.

Our design of synthetic communities emphasized placing every bacterial functional block into at least 2 microbial backgrounds; therefore, we should be able to estimate bacterial effects that are independent of background. In principle, this estimation could be used to design novel synthetic communities with predictable outputs. We found that additivity of bacterial effects could explain most, but not all, of the host phenotypic variation. Therefore, we built 3 different quantitative predictive models capable of capturing different levels of complexity and evaluated their performance. We constructed a simple linear model (LM), a linear model that included pairwise interactions between bacterial functional blocks (INT), and a Neural Network model (Fig 7A and Materials and methods 4). We focused on shoot Pi content, which had the strongest signal-to-noise ratio (SNR) of all plant phenotypes tested (S11 Fig, Materials and methods 4b). To evaluate the predictive performance of each model, we used a form of cross validation in which the data from each synthetic community were held out one at a time, and the remaining synthetic communities were used to train each of the 3 models; those trained models were then used to predict the plant phenotypic output of the held-out consortium. We found that the NN had the lowest cross-validated prediction error of the 3 models and that the difference was statistically significant (*p*-value = 0.0073) (Fig 7B). Neural Networks are popular predictive models because they can capture more complex and nuanced relationships that simpler (linear) models cannot; however, this can come at a cost of reduced interpretability. We performed a sensitivity analysis (Materials and methods 4f) by calculating the effect that changing each variable would have on shoot Pi content according to the NN and the 2 linear models (LM and INT). We found a general agreement between the 3 models; for example, all models showed that Pi level in the media and the presence of negative bacterial blocks had the strongest effect on shoot Pi content, but the NN produces much more fine-grained results, because it is able to predict the change differentially across each condition (Fig 7C).

In order to validate the prediction accuracy of the NN, we chose the 25 bacterial block replacements that were predicted to result in the largest increase in shoot Pi content and experimentally tested whether an increase was produced by these synthetic communities (Fig 7D, S8 Table, and Materials and methods 4g). There was a significant correlation (*ρ* = 0.42, *p*-value = 0.0375) between predicted and observed shoot Pi content changes caused by the bacterial block replacements (Fig 7E). Strikingly, we found that 23 out of 25 bacterial block replacements increased shoot Pi content on average (*p*-value = 0.004; 1,000 permutation tests with synthetic community labels randomly permuted) (S9 Table). Moreover, the improvement in shoot Pi content was statistically significant in 16 out of 25 bacterial block replacements (*p*-value = 0.032; 1,000 permutation tests with synthetic community labels randomly permuted) (S9 Table). Only 1 out of 25 bacterial block replacements significantly decreased Pi content (S9 Table). Again, we noted little correlation between bacterial abundances and their effect on Pi content (S12 Fig and S9 Table). Compared to linear models (LM and INT), the NN had significantly lower prediction errors (*p*-value ≤ 4.65 × 10^−7^) (Fig 7F). In summary, we were able to rationally design novel synthetic communities that lead to predictable plant phenotypic outputs.

## Discussion

While plant responses to stress have been shown to be influenced by associated microbial communities, causal relationships in plant–microbe interactions in a community context and measured phenotypes have proven difficult to establish. This limitation is, in fact, generally true across complex host–microbial interaction systems [12][13][36]. Here, we demonstrate that binary-association assays can inform the design of synthetic bacterial communities that lead to predictable plant phenotypes, an observation seen only once previously, in one animal system [12]. The host phenotypic output of the bacterial synthetic communities was consistent with the output expected from binary interactions. Validation of predictions from an NN confirmed that we could predictably alter certain plant phenotypes by changing the plant’s microbiota membership.

Other phenotypes and host–microbiota systems can likely be studied with this approach. The only requirements are that a microcosm reconstitution system is available and that functional bacterial blocks can be defined, so that synthetic communities that maximize the expected range of phenotypic variance can be constructed (Fig 3A and 3B). In practice, other aspects that are likely to influence the tractability of a system are the functional bacterial diversity and the SNR of the phenotypes being measured. In the case of plant phosphate starvation, we found that bacterial abundances provided no information, and while it is too early to say if this is a general feature, the only other work that directly manipulated a well-defined microbiota to establish its effect on a host phenotype reached a similar conclusion [12]. If this trend continues across other host–microbiota systems, then our approach has the added advantage that strains need not be distinguishable by a specific marker gene. While a simple additive model typically explained more than 50% of the host phenotypic variation, we found value in utilizing an NN approach that was able to capture more complex relationships but remained interpretable and significantly increased our prediction accuracy for novel communities. Our framework is based on empirical validation and thus remains flexible enough to allow for simpler or more complex models, depending on the case.

We achieved high prediction accuracy across an untested set of synthetic communities, thus demonstrating that selecting a subset of the possible communities by partial overlap of bacterial functional block pairs is sufficient to characterize this system. This method requires no design of specific heuristics. Thus, this methodology provides an opportunity to expand the capacity for mechanistic understanding not only of biological networks that control plant phenotypes [37][14] but of other complex ecological systems [12][13][36].

Furthermore, by focusing on block replacements as testable hypotheses, we provide a simple outcome that can be extracted from both linear and nonlinear predictive models. This can guide the next set of experimental designs, thus providing nonlinear methods like deep learning a stronger empirical grounding, rendering them less of a “black-box.”

We demonstrate the utility of our approach by defining mechanistic aspects of the plant phosphate stress response in the presence of combinations of bacterial blocks. We observed that bacteria range in their effect on phosphate content in the plant between severely decreasing and moderately enhancing it. These data are consistent with our previous findings that bacterial interactions with the plant are controlled by negative regulation exerted by the phosphate starvation response on the plant immune system [23]. A similar mechanism was described for the interaction under phosphate-limiting conditions of *A*. *thaliana* with the beneficial fungus *Colletotrichum tofieldiae* [24]. Thus, our results provide additional evidence for mechanisms by which plants and bacteria compete in times of nutritional stress.

The use of multiple bacterial synthetic communities led us to define interesting particular aspects of the phosphate stress response. We observed that certain synthetic communities, such as P2P3 and P1P2, drive an increase in the shoot area compared to axenically grown plants, despite the low shoot Pi content that they engender. These data recapitulate a previous observation [31] on the effect of altering the activity of PHOSPHATE1 (*PHO1*), a gene required for Pi loading into the xylem [38]. These authors found that shoot Pi content could be uncoupled from the developmental responses typically linked to Pi scarcity. We corroborated that reduced shoot growth is not necessarily a direct consequence of Pi limitation. The observations that both bacterial activity and the modulation of *PHO1* expression can uncouple plant phenotypes during the response to low Pi leads us to hypothesize that microbes could interdict PHO1 transport activity, thus modifying Pi translocation from roots to shoots.

Additionally, we found that synthetic community P3N3 uniquely induced a strong transcriptional response to phosphate starvation in the majority of the conditions tested. Plants exposed to this bacterial combination showed a high-level induction of *PHO2*, a ubiquitin-conjugating E2 enzyme required for the degradation of Pi transporters at high Pi [34]. This discovery may explain the intense transcriptional response to Pi starvation caused by this particular synthetic community.

We observed much more variability in the bacterial colonization patterns than in their effects on plant phenotypes. Synthetic communities tended to be dominated by 1 block, but the identity of that block did not correlate with plant phenotype. On the other hand, synthetic communities had remarkably consistent effects on plant phenotypes, and synthetic community membership was sufficient to predict host phenotype. These observations suggest that bacteria–bacteria interactions are critical for microbial community assembly, which is probably a highly dynamic process in which the microbial background determines which bacteria perform well. On the other hand, the effect of bacteria on plant phenotypes is probably due to functional stacking, in which many phenotypically redundant strains with potentially different niches maximize the chance of attaining the desired host phenotypic output. This “lottery model” has been proposed as a major driving mechanism of host colonization by its microbiota at the taxonomic level [39], and it would be interesting to test whether a similar process governs functional assembly.

In conclusion, we provide a general method for the study of various biological host–microbiome systems through rational selection of a tractable subset of the possible combinations of bacteria from a defined collection. We demonstrate that complex relationships among host phenotypes, the microbiota, and the abiotic environment can be captured using deep learning techniques. By testing each block of bacterial strains in multiple synthetic communities, and successfully validating predictions derived from an NN, we demonstrated that it is possible to both infer causality and attain generality when it comes to predicting host phenotypes in this complex system. This approach contributes to the rational design and deployment of microbes to improve responses of hosts to biotic and nutritional stresses.

## Materials and methods

### 1. Exudate preparation and in vitro growth curves

#### a. Seed sterilization

All seeds were surface sterilized with 70% bleach and 0.2% Tween-20 for 8 minutes, followed by 3 rinses with sterile distilled water. This treatment eliminates any seed-borne microbes on the seed surface. Seeds were stratified at 4°C in the dark for 2 days.

#### b. Growth conditions for the exudate preparations

Col-0 seeds were germinated on Johnson medium (KNO_3_ [0.6 g/L], Ca(NO_3_)_2_.4H_2_O [0.9 g/L], MgSO_4_.7H_2_O [0.2 g/L], KCl [3.8 mg/L], H_3_BO_3_ [1.5 mg/L], MnSO_4_.H_2_O [0.8 mg/L], ZnSO_4_.7H_2_O [0.6 mg/L], CuSO_4_. 5H_2_O [0.1 mg/L], H_2_MoO_4_ [16.1 μg/L], FeSO_4_.7H_2_O [1.1 mg/L], Myo-Inositol [0.1 g/L], MES [0.5 g/L], pH 5.6–5.7, 1% bacto-agar [BD, Difco]), 0.5% sucrose, solidified with 0.6% agar and supplemented or not with 1 mM Pi, in a horizontal position (approximately 160 seedlings per plate). After 7 days of growth, seedlings were transferred to a 12-well plate. Each well was filled with 3 mL of liquid Johnson medium and between 50 and 60 seedlings. Seedlings were transferred to the opposite Pi concentration from the germination conditions (i.e., plants that were initially grown in 1 mM Pi were transferred to liquid medium with no supplementation of Pi and vice versa).

Plants were grown in liquid media with agitation (30 rpm) for 24 hours in a growth chamber, in a 16-hour light/8-hour dark regime (24°C/21°C). In these conditions, we incubated additional 12-well plates containing Johnson medium alone, supplemented with or without 1 mM Pi. We used these samples as controls for the next experiments. Liquid supernatants containing root exudate and control samples were collected, filtered (0.22 μm), and used for the next experiments.

#### c. Screening of the bacterial strain collection in different plant root exudates

All bacterial strains used in this work were isolated from roots of Brassicaceae grown in 2 well-studied wild soils from North Carolina, US [32][40][28]. For the screening of the bacterial strain collection in different plant root exudates, bacteria from −80°C glycerol stocks were grown on LB plates at 28°C. A single colony was then inoculated in 200 μL of 2xYT medium (16 g/L Tryptone, 10 g/L yeast extract, 5 g/L NaCL, about 5.5 mM Pi) in a 96-well polystyrene plate (Costar) and covered with a breathable sealing film (Excel) to prevent contamination. Bacterial cultures were grown with agitation at 28°C. After 24 hours, all cultures were diluted 1/10 into the different plant exudates and control media and grown at 28°C with agitation. Exudate and media samples without bacteria were included as controls of contamination (uninoculated controls). To analyze the growth curves of the different isolates, the OD at 600 nm (OD_600nm_) was measured every 3 hours during the day and every 14 hours during the night for 3 days using a microplate reader (Tecan, GENios).

#### d. Quantification of primary metabolites in plant root exudates

Primary metabolites profiling was performed using an ALEX-CIS GCTOF MS in the NIH West Coast Metabolomics Center (University of California, Davis, CA). Plant root exudates and control samples were extracted following Fiehn et al. [41]. 30-μL aliquots of each sample were extracted by 1 mL of degassed acetonitrile:isopropanol:water (3:3:2, v/v/v) at –20°C, centrifuged, and decanted, with subsequent evaporation of the solvent to complete dryness. A cleanup step with acetonitrile/water (1:1) removed membrane lipids and triglycerides. The cleaned extracts were aliquoted into 2 equal portions and the supernatants were dried down again. Internal standards C08-C30 FAMEs were added, and the samples were derivatized by methoxyamine hydrochloride in pyridine and subsequently by N-methyl-N-trimethylsilyltrifluoroacetamide for trimethylsilylation of acidic protons. Data were acquired using the chromatographic parameters published in Fiehn et al. [42]. We used a column from Restek corporation: rtx5Sil-MS (30 m length × 0.25 mm internal diameter, with 0.25-μm film made of 95% dimethyl/5% diphenylpolysiloxane. Helium was used as the mobile phase with a column temperature of 50–330°C and a flow rate of 1 mL min^−1^. 0.5 μL of sample was injected with 25 splitless time into a multi-baffled glass liner at 50°C ramped to 250°C by 12°C s^−1^.

Mass spectrometry parameters were used as follows: a Leco Pegasus IV mass spectrometer was used with unit mass resolution at 17 spectra s−1 from 80–500 Da at −70 eV ionization energy and 1,800 V detector voltage with a 230°C transfer line and a 250°C ion source.

Raw data were normalized according to NIH West Coast Metabolomics Center (University of California, Davis, CA) quality standards.

#### e. Analysis of the primary metabolites in plant root exudates

For the metabolite analysis, we standardized the metabolite abundances by dividing the abundance of each metabolite by the total abundance in each sample. To visualize the general patterns in our dataset, we independently applied hierarchical clustering on metabolites and samples. We clustered samples according to their Bray-Curtis dissimilarities, which were calculated with function vegdist in the R vegan package [43]. Metabolites were clustered according to a Pearson correlation dissimilarity matrix, computed using the formula 1−cor(x) via the function cor in R [44]. In both cases, the hclust function in R was used with the method “ward.D” [44] to create the corresponding dendrograms. The resulting dendrograms were visualized together with the metabolite relative abundances using the function heatmap.2 from the package gplots [45], with the parameter scaling by row, which corresponded to metabolites.

We calculated the log(fold-change) for each metabolite in each exudate sample, in relation to its respective media control sample (Table 1).

**Table 1:** The table shows the log(fold-change) schema of relationships between exudate samples and control samples.

Exudate Samples	Control Samples
Exudate −/−	Control −
Exudate +/−	Control −
Exudate +/+	Control +
Exudate −/+	Control +

Then, for each sample, we calculated the log_2_(fold-change) of each metabolite with respect to its control by subtracting the log2-transformed mean relative abundance of that metabolite in the control samples to the log2-transformed relative abundance value of the metabolite in a given sample.

To visualize the matrix derived from the log(fold-change) calculation, we independently applied hierarchical clustering on the metabolites and on the samples. We clustered exudate samples based on the euclidean distance calculated with the dist function in R [44]. Metabolites were clustered according to a Pearson correlation dissimilarity matrix, computed using the formula 1−cor(x) via the function cor in R [44]. In both cases, the hclust function in R was used with the method “ward.D” [44] to create the corresponding dendrograms. The resulting dendrograms were visualized together with the metabolite log_2_(fold-changes) using the function heatmap.2 from the package gplots [45], with the parameter scaling by row, which corresponded to metabolites.

All the scripts developed for this analysis are available at: https://github.com/isaisg/primary_metabolites_phosphate.

#### f. Isolate QC and feature extraction

Bacterial growth curves were quality filtered by removing strains that had growth profiles that were highly similar to uninoculated samples. All the following operations were done with functions available via the PGCA R package (https://github.com/surh/PGCA). For each strain and condition, the median growth curve was obtained by calculating the median OD_600nm_ per time point. The 4 resulting growth curves (for 4 conditions) were concatenated and grouped by hierarchical clustering based on their correlation distance according to the formula *d*_*xy*_ = 1 − *ρ*_*xy*_, where *ρ*_*xy*_ is the Pearson correlation coefficient between strains *x* and *y*. The resulting clustering dendrogram was cut at a height of 0.5, based on visual inspection. Clusters that had more than 40% uninoculated controls were discarded together with any strains that fell within them. The remaining uninoculated controls were also discarded. We then calculated the AUC for each strain and condition by adding the median OD_600nm_ per time point using PGCA (https://github.com/surh/PGCA).

Four other growth curve features were extracted for each strain and condition. First, a moving average filter smoothed all data. For this, we applied a regular moving average filter with window size of 5 and step size of 1 per individual replicate. We defined the following metrics for each strain under each condition using the smoothed growth curves:

1. Maximum OD (MAX). MAX is the maximum OD reached among all 4 replicates and all time-series points.
2. Mean density over the last 3 measurements (L3M). L3M is used to give an approximate measurement of the OD at saturation by averaging the last 3 measurements in the time series over all replicates.
3. Mean time to reach half maximum (HMT). The time for a strain to reach half maximum is the time stamp of the earliest sample in the time series data that has an OD greater than or equal to half of the MAX. We take the mean over all replicates to calculate HMT. We use HMT to show how fast the strain is growing.
4. Maximum growth rate (MGS). The growth rate at a particular time point is calculated by (*log*_2_ *x*_*tk*+1_ − *log*_2_ *x*_*tk*_)/(*t*_*k*+1_ − *t*_*k*_), where *x*_*tk*_ represents the OD of the strain at time *t*_*k*_. MGS is the maximum growth rate among all replicates and all time points.

Each of these features was calculated for the 4 conditions: Johnson media supplemented or not with Pi (+Pi or −Pi, respectively); Johnson media supplemented with root exudates from 2 short and complementary nutritional Pi transitions (+Pi_−Pi or −Pi_+Pi). Furthermore, we also normalized each growth curve by their control by calculating log_2_(fold-change) on each specific feature between −Pi_+Pi and +Pi (MLF), and +Pi_−P and −Pi (PLF).

#### g. Isolate growth-curve clustering and selection for binary-association assays

In general, several features analyzed contain redundant information (S2A Fig). This redundancy allowed us to select 1 parameter, the AUC, as an aggregate measure of bacterial performance for further analysis.

For grouping strains according to their growth performance, each strain’s AUC was log transformed and then standardized per condition by subtracting its mean and dividing by the standard deviation. Hierarchical clustering was used with the euclidean distance and the complete linkage method in R [44]. In order to select strains to test in binary-association assays, an ANOVA model was fit on each strain, using the AUC values as dependent variables and condition (media) as the only independent variable. We calculated the *R*^*2*^, which indicated which proportion of the variation in the bacterial growth performance (AUC) is attributable to media—in other words, how much of the bacterial growth is affected by plant exudates and phosphate levels. We prioritized testing multiple strains per cluster that had the highest *R*^*2*^ values. The code and data to perform these analyses are bundled in the R package (wheelP) [https://github.com/surh/wheelP].

#### h. Phylogenetic signal analyses

For all strains with an available Sanger generated 16S rRNA gene sequence (395 strains out of 440), we used MUSCLE [46] to perform a multiple sequence alignment with default parameters. We then filtered out positions that had more than 99% gaps as well as the top 10% most entropic sequences using QIIME [47]. The resulting filtered alignment was used to build a maximum likelihood tree with FastTree [48], using midpoint rooting.

We standardized all the phenotypes to allow for simultaneous visualization and easier comparison. We used the phylosig function from the phytools R package [49] to test Pagel’s *λ* for phylogenetic signal. Results were visualized with the ggtree R package [50]. The code and data to perform these analyses are bundled in the R package (wheelP) [https://github.com/surh/wheelP].

### 2. Binary associations

#### a. Growth conditions for plant–bacteria binary-association assays

Plants were germinated in axenic condition on Johnson medium plus 0.5% sucrose with 1 mM Pi, about 5 μM Pi (traces of Pi from the agar [Difco]), or supplemented with 1 mM phosphite in a vertical position for 6 days. Seedlings (10 seedlings per plate) were then transferred to 30 μM Pi and 100 μM Pi media (without sucrose), alone or with the bacterial culture, at 10^5^ c.f.u/mL of medium for another 7 days. We harvested shoot samples for Pi quantification. Plants were grown in a growth chamber in a 16-hour light/8-hour dark regime (24°C/21°C). We used 3 replicas per condition and bacterial strain; the experiment was repeated once.

To confirm that plants germinated in different Pi regimens or with phosphite differentially activated the phosphate starvation response, plants carrying the phosphate stress reporter construct *IPS1*:*GUS* [22] were grown in Johnson medium containing 1 mM Pi, 1 mM phosphite or traces of Pi (about 5 μM Pi). After 7 days, the expression of the reporter constructs *IPS1*:*GUS* was followed by GUS staining (S3C Fig).

To study the colonization of the plant shoot by root-inoculated bacteria (Fig 2D and S3A Fig), we germinated Col-0 on Johnson medium 1 mM Pi, about 5 μM Pi (traces of Pi from the agar [Difco]), or 1 mM phosphite for 7 days. Seedlings (5 seedlings per plate) were then transferred to 2-compartment plates for a week. In this system, root and shoot were placed in different compartments separated by a plastic barrier to prevent microbe diffusion through the medium. The root compartment was previously filled with Johnson medium 30 μM Pi solidified with 1% agar containing bacteria, and the shoot compartment was filled with a solution of agar (water + 1% agar). Plants were grown in a growth chamber in a 16-hour light/8-hour dark regime (24°C/21°C). We harvested shoot and root separately, and bacteria accumulation in shoots, roots, and root-surrounding agar was analyzed in mock-inoculated plants and plants colonized by bacteria. We used 5 replicas per condition and bacterium; the experiment was repeated once with similar results.

#### b. Bacterial culture for binary association and synthetic community experiments

For binary association and synthetic community experiments, a single colony was inoculated in 4 mL of 2xYT medium in a test tube. Bacterial cultures were grown at 28°C with agitation overnight. Cultures were then rinsed with a sterile solution of 10 mM MgCl_2_ followed by a centrifugation step at 2,600 g for 8 minutes. This process was repeated twice to eliminate any additional nutrient supplementation in the media. The OD_600nm_ was measured and, assuming that 1 OD_600nm_ unit is equal to about 10^9^ c.f.u/mL, we equalized individual strain concentration to a final value of 10^5^ c.f.u/mL of medium. The medium was cooled (to 40–44°C) near the solidification point and then the bacterial strain or the bacteria strain mix was added to the medium with agitation.

#### c. Re-isolation and quantification of bacteria using c.f.u’s

For bacterial colonization analysis in binary-association assays, roots, shoots, and agar samples were harvested and weighed. Roots and shoots were rinsed 3 times with sterile distilled water to remove agar particles and then placed in a sterile tube with 200 μL of 10-mM MgCl_2_ and glass beads. Plant material and agar samples were then crushed using 2 cycles of 30 seconds, frequency 30 s^−1^, in a TissueLyser II (Qiagen). These samples were serially diluted, plated on LB, and c.f.u/mL of harvested samples were determined.

#### d. Determination of phosphate concentration in the plant shoot

The method of Ames [51] was used to determine the free phosphate concentration in the shoots of seedlings grown on different Pi regimens and treatments.

#### e. Analysis of bacterial effect on shoot phosphate accumulation

We identified which strains had a statistically significant effect on shoot phosphate accumulation. We compared the phosphate content between plants treated with a given strain and axenically grown plants from the same experiment. To determine significance, we log transformed the phosphate content and used an ANOVA model in R [44] with terms for bacterial treatment and biological replicate. Each of the 4 phosphate conditions was analyzed independently. By testing the bacterial treatment term in the model, we determined whether the effect was significant. We corrected all the obtained *p*-values using the Benjamini-Hochberg method [52], and considered significant all effects that had a *q*-value < 0.1. The code and data to perform this statistical analysis are bundled in the R package (wheelP) [https://github.com/surh/wheelP].

#### f. Phylogenetic signal analyses

To facilitate the simultaneous visualization and to control for batch effects, we scaled all the bacterial effects with respect to the no bacteria controls in the same experiments. We did this by using the results from the ANOVA analysis (Materials and methods 2e) as input for the phylogenetic analysis. These ANOVA results represent the log(fold-change in Pi content, with respect to no bacteria) caused by each strain. We used the phylosig function from the phytools R package [49] to test Pagel’s *λ* for phylogenetic signal. We utilized the same tree that we constructed for the phylogenetic analysis of in vitro growth (Materials and methods 1h). 177 out of 183 strains were present in this tree. Results were visualized with the ggtree R package [50]. The code and data to perform these analyses are bundled in the R package (wheelP) [https://github.com/surh/wheelP].

### 3. Synthetic community experiments (including 16S analysis and RNA-Seq)

#### a. Growth conditions for synthetic community experiments

For synthetic community experiments, plants were germinated in axenic condition on Johnson medium plus 0.5% sucrose with 1 mM Pi or about 5 μM Pi (traces of Pi from the agar [Difco]) in a vertical position for 6 days, then transferred (10 seedlings per plate) to 30 μM Pi and 100 μM Pi media (without sucrose), alone or with the synthetic community, at 10^5^ c.f.u/mL of medium (see Materials and methods 2b) for another 7 days. Plants were grown in a growth chamber in a 16-hour light/8-hour dark regime (24°C/21°C). Plant material (root) was collected for 16S profiling, for transcriptional analysis (seedlings), and shoots were used for Pi quantification. This experiment was repeated once with similar results, and each repetition included 3 biological replicates (10 plants each) per condition and per synthetic community analyzed.

#### b. Determination of plant morphological parameters

Primary root length was measured using ImageJ [53], and shoot area and total root network were measured with WinRhizo [54].

#### c. Bacterial DNA extraction

For bacterial colonization analysis using 16S, roots were surface sterilized with freshly made 10% household bleach with 0.1% Triton-X100 for 12 minutes. Following the bleaching, roots were rinsed once in sterile distilled water, then placed in 2.5% sodium thiosulfate to neutralize the bleach for 2 minutes, and rinsed once more with sterile distilled water. Roots were then freeze-dried and powdered in a 2-mL tube with glass beads using the MPBio FastPrep for 20 seconds at 4.0 m/s. These samples were used for DNA extraction using 96-well format MoBio PowerSoil kit (SDS/mechanical lysis).

To quantify bacteria from agar samples, a freeze and squeeze protocol was used. Syringes with a square of sterilized miracloth on the bottom were completely packed with agar samples and kept at −20°C for a week. Samples were thawed at room temperature and syringes were squeezed gently into 50-mL tubes. Samples were centrifuged at max speed for 20 minutes, and most of the supernatant was discarded. The remaining 1–2 mL of supernatant containing the pellets was moved into clean microfuge tubes. Samples were centrifuged again, supernatants were removed, and pellets were used for DNA extraction with the 96-well format MoBio PowerSoil kit (SDS/mechanical lysis). DNA was extracted simultaneously for both agar and root samples. We performed randomization of the sample order using a mechanical method.

#### d. 16S rRNA gene library preparation

We amplified the V3–V4 regions of the bacterial 16S rRNA gene using primers 338F (5′-ACTCCTACGGGAGGCAGCA-3′) and 806R (5′-GGACTACHVGGGTWTCTAAT-3′). Libraries were created using a modified version of the Lundberg et al. [55]. The molecule-tagging step was changed to an exponential amplification to account for low DNA yields, with the following reaction and temperature cycling conditions (Table 2).

**Table 2:** Reaction and temperature cycling conditions used for the exponential amplification step during the library preparation.

**Reaction**	
**Volume**	**Reagent**
5 μL	Kapa Enhancer
5 μL	Kapa Buffer A
1.25 μL	5 μM 338F
1.25 μL	5 μM 806R
0.375 μL	mixed PNAs (1:1 mix of 100 μM pPNA and 100 μM mPNA)
0.5 μL	Kapa dNTPs
0.2 μL	Kapa Robust Taq
8 μL	dH_2_O
5 μL	DNA
**Temperature cycling**	
**Temperature**	**Time**
95°C	60 seconds
24 cycles of	
95°C	15 seconds
78°C (PNA)	10 seconds
50°C	30 seconds
72°C	30 seconds
4°C	until use

Abbreviations: dH_2_O, distilled water; dNTP, deoxynucletide; PNA, Peptide Nucleic Acid; mPNA, PNA targeting *A*. *thaliala* mitochondrial 16S gene sequences; pPNA, PNA targeting *A*. *thaliana* plastid 16S gene sequences; Taq, Taq polymerase.

Following PCR cleanup to remove primer dimers, the PCR product was indexed using the same reaction and 9 cycles of the cycling conditions described in Lundberg et al. [55]. Sequencing was performed at The University of North Carolina at Chapel Hill, Chapel Hill, NC, on an Illumina MiSeq instrument, using a 600-cycle V3 kit. The raw data from these sequencing experiments are available at the ENA Sequence Read Archive (accession number **PRJEB22060**).

#### e. 16S rRNA profiling sequence processing and analysis

Synthetic community sequencing data were first processed with MT-Toolbox [56]. Categorizable reads from MT-Toolbox (i.e., reads with correct primer and primer sequences that successfully merged with their pair) were quality filtered with Sickle by not allowing any window with a Q-score under 20, and trimmed from the 5′ end to a final length of 350 bp. The resulting sequences were matched to a reference set of the strains in the synthetic community generated from Sanger sequences and *A*. *thaliana* organellar sequences. Sequence mapping was done with USEARCH7.1090 [57] with the option “-usearch global” at a 98.5% identity threshold (which translates to 4 mismatches for our sequence length). Sequences from each sample were only mapped to the expected sequences, given the synthetic community block composition. Matching sequences were used to produce an abundance table that was input into AMOR [58]. Host nuclear and organellar-derived sequences were filtered out, and samples that had less than 400 reads were removed. The resulting table was collapsed by strain order or strain block in AMOR [58]. Aggregation was performed by arithmetic addition of all the counts belonging to a given group. Taxonomic and block-level distributions were visualized with the phylogram function in AMOR [58], and constrained ordination was performed with vegan’s capscale function [43] and visualized with ggplot2 [59]. The code and data to perform this analysis are bundled in the R package (wheelP) [https://github.com/surh/wheelP].

#### f. Plant RNA isolation

Total RNA was extracted from *A*. *thaliana* seedlings according to Logemann et al. [60]. Frozen seedlings were pulverized in liquid nitrogen. Samples were homogenized in 400 μl of Z6-buffer; 8 M guanidinium-HCl, 20 mM MES, 20 mM EDTA pH 7.0. Following the addition of 400 μL phenol:chloroform:isoamylalcohol, 25:24:1, samples were vortexed and centrifuged (20,000 g, 10 minutes) for phase separation. The aqueous phase was transferred to a new 1.5-mL tube and 0.05 volumes of 1 N acetic acid and 0.7 volumes 96% ethanol were added. The RNA was precipitated at −20°C overnight. Following centrifugation (20,000 g, 10 minutes, 4°C), the pellet was washed with 200 μl sodium acetate (pH 5.2) and 70% ethanol. The RNA was dried and dissolved in 30 μL of ultrapure water and stored at −80°C until use.

#### g. RNA-Seq library construction

We performed an RNA-Seq experiment to evaluate the effect of the different synthetic communities on the transcriptional profile of *A*. *thaliana* seedlings. Wild-type Col-0 was germinated under Pi-sufficient and Pi-depleted conditions for 6 days and then transferred to 30 μM Pi and 100 μM of Pi for another 7 days, with or without bacteria. At this point, seedlings were harvested for RNA-Seq. This experiment was repeated once, and each repetition included 3 biological replicates per genotype per condition per synthetic community analyzed. Raw reads and read counts are available at the NCBI Gene Expression Omnibus under accession number **GSE102248**.

Illumina-based mRNA-Seq libraries were prepared from 1 μg RNA. Briefly, mRNA was purified from total RNA using Sera-mag oligo(dT) magnetic beads (GE Healthcare Life Sciences) and then fragmented in the presence of divalent cations (Mg^2+^) at 94°C for 6 minutes. The resulting fragmented mRNA was used for first-strand cDNA synthesis using random hexamers and reverse transcriptase, followed by second-strand cDNA synthesis using DNA Polymerase I and RNAseH. Double-stranded cDNA was end repaired using T4 DNA polymerase, T4 polynucleotide kinase, and Klenow polymerase. The DNA fragments were then adenylated using Klenow exo-polymerase to allow the ligation of Illumina Truseq HT adapters (D501–D508 and D701–D712). All enzymes were purchased from Enzymatics. Following library preparation, quality control and quantification were performed using a 2100 Bioanalyzer instrument (Agilent) and the Quant-iT PicoGreen dsDNA Reagent (Invitrogen), respectively. Libraries were sequenced using Illumina HiSeq2500 sequencers to generate 50-bp single-end reads.

#### h. RNA-Seq sequence processing and analysis

Initial quality assessment of the Illumina RNA-Seq reads was performed using the FASTXToolkit (http://hannonlab.cshl.edu/fastx_toolkit/index.html). Cutadapt [61] was used to identify and discard reads containing the Illumina adapter sequence. The resulting high-quality reads were then mapped against the TAIR10 *A*. *thaliana* reference genome using Tophat [62], with parameters set to allow only one mismatch and discard any read that mapped to multiple positions in the reference. The Python package HTSeq [63] was used to count reads that mapped to each one of the 27,206 nuclear protein-coding genes. Raw sequencing data and read counts are available at the NCBI Gene Expression Omnibus accession number **GSE102248**.

For expression of the phosphate starvation response core transcriptional markers and the clustering analysis, we converted the count table into a table of reads per kilobase per million (RPKM) and standardized these values per gene by subtracting the mean gene expression and dividing by the standard deviation of each gene’s expression. Hierarchical clustering was performed with the R function hclust [44] using the complete linkage method. Gene ontology enrichment analysis was performed on the PlantGSEA [64] online platform.

For specific hypothesis tests, we used edgeR [65] to fit a quasi-likelihood negative binomial model with the function glmQLFit, after estimating tagwise dispersion parameters. We then applied a quasi-likelihood ratio test with the function glmQLFTest in edgeR [65] using different sets of contrast. Our model specification included indicator terms for each bacterial block, as well as terms for phosphate condition, biological replicate, and interaction between phosphate condition and each block. This was defined according to the following formula:

$$\begin{array}{c} \begin{array}{c} \text{Expression}\;=\;\text{P}1\;+\;\text{P}2\;+\;\text{P}3\;+\;\text{I}1\;+\;\text{I}2\;+\;\text{I}3\;+\;\text{N}1\;+\;\text{N}2\;+\;\text{N}3+\\ \text{Phosphate}\;+\;\text{Experiment}\;+ \end{array}\\ \text{P}1*\text{Phosphate}\;+\;\text{P}2*\text{Phosphate}\;+\;\text{P}3*\text{Phosphate}\;+\\ \text{I}1*\text{Phosphate}\;+\;\text{I}2*\text{Phosphate}\;+\;\text{I}3*\text{Phosphate}\;+\\ \text{N}1*\text{Phosphate}\;+\;\text{N}2*\text{Phosphate}\;+\;\text{N}3*\text{Phosphate} \end{array}$$

The first 9 terms are indicator variables for the bacterial blocks of the synthetic communities that take the value of 1 when that block is present. Phosphate has 4 levels that correspond to the 2 × 2 phosphate condition experimental design. The experiment has 5 levels that correspond to independent biological replicates of the synthetic community experiments (each community was in 2 biological replicates). A total of 41 coefficients, including intercept, are generated from this design. The definitions of the contrasts used for the different hypotheses are bundled in the R package (wheelP) [https://github.com/surh/wheelP].

The code and data to perform this analysis are bundled in the R package (wheelP) [https://github.com/surh/wheelP].

#### i. Design of the synthetic communities

Due to the fact that the majority of the strains analyzed in binary interaction had a negative effect on shoot Pi accumulation, we first identified strains that had a significant positive effect (*q*-value < 0.1 from ANOVA) in the 2 conditions that ended on 100 μM phosphate. We found 26 strains and we labeled them as positive strains. Many strains are negative in 2 conditions or more, so we first identified strains that had a statistically significant (*q*-value < 0.1 from ANOVA) negative effect on shoot phosphate accumulation in at least 3 of the 4 conditions. We then identified strains that had a statistically significant negative effect in at least 2 conditions, but with higher statistical confidence (*q*-value < 0.05). We removed 2 strains that did not come from our Brassicaceae cultivation efforts in 2 natural soils [32][40] (strains *Pseudomonas fluorescens* WCS417r and *Rhizobium sp*. R219). We combined the 2 sets of negative strains and obtained 26 strains that we termed negative strains. We finally identified strains that had no statistically significant effect (*q*-value > 0.1 from ANOVA) on phosphate accumulation across all conditions, and from this set we randomly and computationally selected 26 strains that we termed the Indifferent strains. We finally calculated the mean effect of each strain on shoot phosphate accumulation by averaging the coefficients from the ANOVA in all conditions. We sorted the strains within each group according to that mean, and we divided each group into 3 blocks of bacteria by taking groups of 9, 9, and 8 strains (9 + 9 + 8 = 26). The bacterial blocks defined this way are the basis for the synthetic community design. The code and data to perform this statistical analysis are bundled in the R package (wheelP) [https://github.com/surh/wheelP].

We tested a set of 14 partially overlapping synthetic communities. Each of the synthetic communities was made of a combination of 2 bacterial blocks. We made 9 synthetic communities by combining adjacent blocks (i.e., blocks that are next to each other when sorted by their mean effect). Each of these 9 communities is represented as an outer arc in Fig 3B, and they are composed mostly of strains that have similar effects on shoot phosphate accumulation when tested in binary association; however, they represent the widest possible range of mean effects, and so we expect they might produce different plant phenotypes. We constructed another 5 synthetic communities, which are represented as inner arcs in Fig 3B that represent extra combinations of the most extreme blocks (i.e., P1 and N3), in order to test how strong their effects are in a variety of backgrounds.

#### j. Estimating block additivity

To determine the level of consistency of bacterial block effects on different plant phenotypes, we first compared plants inoculated with each synthetic community versus axenically grown controls. We then estimated the main effects of each block using multiple regression and compared the coefficients obtained from both methods. Phosphate content and shoot area measurements were log transformed to reduce heteroscedasticity, and thus the coefficients from this analysis should be interpreted as log(fold-change) between inoculated plants and axenic controls. The LMs adequately represented measurements from primary root elongation and total root network, and so coefficients for these 2 phenotypes should be interpreted as the difference between inoculated plants and axenic controls. Appropriateness of the LMs and the need and correctness of transformation was determined via inspection of residual plots. Only the 14 original synthetic communities were included in the analysis.

To estimate synthetic community effects, we fit a linear model per phenotype and condition, with only the samples of one synthetic community at a time, plus the axenic controls performed in the same experiment. We had only bacterial treatment and experiment variables, according to the following formula:

Phenotype=SynCom+Experiment

The resulting SynCom coefficient was denoted as the “measured” synthetic community effect: To find the expected phenotypic effect of each synthetic community, we first estimated each block’s additive (main) effect. We did this by fitting all the data from each media condition into 1 linear model containing only terms for each block and experiment as independent variables, using the following formula:

Phenotype=P1+P2+P3+I1+I2+I3+N1+N2+N3+Experiment

Each of the block variables is encoded as an indicator variable, and each has the value of “1” when the corresponding block is present and “0” when the corresponding block is absent. We finally obtained the “predicted” community effect by arithmetically adding the coefficients for the 2 blocks that make each community. To compare the effects of both synthetic communities and blocks together, we rescaled them by dividing them by the standard deviation of all coefficients from the same phenotype in the same condition (i.e., by column). The code and data to perform this statistical analysis are bundled in the R package (wheelP) [https://github.com/surh/wheelP].

#### k. Estimating block additivity as a function of relative abundance

In order to determine if bacterial relative abundances are correlated with host phenotypes, we repeated the analysis above (Materials and methods 3j) but substituted the indicator variables of each block for the relative abundance of that block.

Because the relative abundance data are obtained from a pool of plants and require destructive sampling, we averaged all the measurements from each independent biological replicate and used those averages as input for the analysis. This effectively reduces the number of observations and increases the standard errors. In order to achieve a fair comparison, we also recalculated the purely additive contributions of each block (without considering abundance), and we equalized the norm of the coefficients between the 2 versions of the analysis. Results are presented in S7 Fig, and the code and data to perform this statistical analysis are bundled in the R package (wheelP) [https://github.com/surh/wheelP].

### 4. Neural network (including validation experiment)

#### a. Growth conditions for the NN validation experiments

Plants were germinated in axenic condition on Johnson medium plus 0.5% sucrose without supplementation of Pi in a vertical position for 6 days, then transferred to 30 μM Pi medium (without sucrose), alone or with the synthetic communities at 10^5^ c.f.u/mL of medium for another 7 days. Plants were grown in a growth chamber in a 16-hour light/8-hour dark regime (24°C/21°C). Plant material (shoot) was collected for Pi quantification and roots were used for 16S profiling. This experiment was repeated once.

#### b. Estimating SNR for each phenotype

To determine the feasibility of predicting the phenotypic effect of novel communities, we calculated the SNR for each phenotype. There are 120 different input conditions per phenotype (experiment, Pi condition, and synthetic community input). For each condition, there are 3 replicate measurements for plant shoot area, total root network length, and Pi content and around 20–30 replicates for primary root elongation. Let **x**_**c**_ = [x_1c_, x_2c_, …, x_rc_] be the measurement of all replicates for a particular phenotype under condition c. We define the signal of a specific input condition as the mean measurement over all replicates in that condition.

$${Signal}_{c}\;=\;\frac{1}{r}\;\sum_{i\;=\;1}^{r}x_{ic}$$

The signal variance is the variance of signals over all input conditions.

We define the noise for a specific input condition as the residual between the mean and the actual measurement of the replicates:

$${Noise}_{ic}\;=\;x_{ic}-\;{Signal}_{c}$$

The noise variance is the variance of the noise over all input conditions and replicates (Table 3).

**Table 3:** The table shows SNRs associated with the plant phenotypes analyzed.

Phenotypes	Shoot Area	Root Length	Pi content	Primary Root Elongation
**SNR**	3.8907	3.2276	**13.7852**	2.0980

The SNR is defined as the ratio between signal variance and noise variance. In bold is the SNR value for shoot Pi content that has the strongest SNR of all plant phenotypes tested. The signal variance and noise variance are shown in S11 Fig.

Abbreviations: Pi, phosphate; SNR, signal-to-noise-ratio.

#### c. Neural network construction

We focused on shoot phosphate accumulation, which had the largest SNR of all phenotypes and serves as a bedrock test for our approach. Input data consist of a biological replicate ID b ∈ {1,2}, a technical replicate ID r ∈ {1,2,3}, pretreatment indicator p ∈ {−Pi, +Pi} and phosphate-level q ∈ {30 μM, 100 μM}, and a SynCom setting S ⊆ {P1,P2,P3,I1,I2,I3,N1,N2,N3}. We call the combination of p and q the phosphate condition. A design is a combination of phosphate condition and synthetic community. An input condition is a combination of design and biological replicate ID. Let *z*_*b*,*p*,*q*,*S*,*r*_ be the standardized Pi-content measurement under biological replicate *b*, technical replicate *r*, pretreatment *p*, phosphate-level *q*, and SynCom setting *S*. The mean of Pi content across 3 technical replicates is

$$y_{b,p,q,S}\;=\;\frac{1}{3}\sum_{r\;=\;1}^{3}z_{b,p,q,S,r}$$

and its variance is

$$v_{b,p,q,S}\;=\;Var(z_{b,p,q,S,1},z_{b,p,q,S,2},z_{b,p,q,S,3})$$

An input ***x***_*b*,*p*,*q*,*S*_ is constructed as a binary vector of length 12 (Table 4). A model learns a function $f(x_{b,p,q,S})\;=\;{\hat{y}}_{b,p,q,S}$ for the prediction of mean Pi content.

**Table 4:** The table shows the variables used to construct the input as a binary vector.

Feature name	Biological replicate ID b	Pretreatment p	Phosphate level q	Synthetic community bacterial block indicator S
x = 0 or 1	b = 1 or 2	p = −Pi or +Pi	q = 30 uM or 100 uM	Corresponding bacterial block is absent/present in this design

#### d. Linear model (LM) and linear model with interaction features (INT)

An LM has the following form:

$$f_{LM}(x)\;=\;\beta +\;x^{T}w,$$

where *β* is the bias term. ***w*** is the linear coefficients for each features in ***x***. If interaction features are considered, we have the following form:

$$f_{INT}(x)\;=\;\beta +\;x^{T}w+\sum_{i\;=\;1}\sum_{j\;=\;i+1}x_{i}x_{j}{ϴ}_{i,j},\;$$

where **ϴ** is an upper triangular matrix for which diagonal entries are 0, and each entry *ϴ*_*i*,*j*_ indicates the linear coefficient for the interaction term *x*_*i*_ *x*_*j*_. Compared to LM, INT is able to capture condition-specific influences. For example, a synthetic community can have different impact on Pi content under different phosphate conditions. Elastic net regularization [66] is used in learning the parameters for both linear model and linear model with interaction features. An elastic net regularization of LM and INT yields the following optimization objective:

$$\begin{array}{l} LM:\beta^{*},w^{*}=arg\underset{\beta ,w}{\text{min}}\sum_{t}{\left(y_{t}-x_{t}^{T}w-\beta \right)}^{2}+\lambda_{1}\left|w\right|+\lambda_{2}\|w{\|}_{2}^{2},\\ INT:\beta^{*},w^{*},{ϴ}^{*}=arg\underset{\beta ,w,ϴ}{\text{min}}\sum_{t}{\left(y_{t}-x_{t}^{T}w-\beta -\sum_{i=1}\sum_{j=i+1}x_{t,i}x_{t,j}{ϴ}_{i,j}\right)}^{2}+\\ \lambda_{1}\left(\left(w\right)+\left|ϴ\right|\right)+\lambda_{2}\left({\left(w\right)}_{2}^{2}+{\left(ϴ\right)}_{2}^{2}\right), \end{array}$$

where λ_1_, λ_2_ are the elastic net regularization penalty weights.

#### e. Neural network (NN)

We used a multilayer feed-forward NN, a typical framework in the deep NN structure family, in which input data are combined and transformed nonlinearly through multiple layers of artificial neurons [67]. An NN contains an input layer, an output layer, and L hidden layers. The **depth** of the network is the number of hidden layers, and the **width** is the number of nodes in each hidden layer. For convenience, the input layer is defined as **h**_**0**_(**x**) = **x**, and the output of lth hidden layer is defined as **h**_l_(**x**). The number of nodes in layer l is m_l_. The input into the lth layer of the network is defined as

$$a_{l}\left(x\right)=\;h_{l-1}{\left(x\right)}^{T}W_{l}+\;\beta_{l},$$

where **W**_**l**_ is a real value weight matrix of m_l-1_ by m_l_, and **β**_**l**_ is a vector of length m_l-1_. The output of lth hidden layer is

$$h_{l}(x)\;=\;leakyReLU(a_{l}(x)),$$

where leaky Rectified Linear Unit [68] activation function is:

$$\begin{array}{cc} leakyReLU\left(x\right)= & \{\begin{array}{ll} x, & if\;x\;\geq 0\\ 0.01x, & \text{otherwise}. \end{array} \end{array}$$

Finally, a linear output layer is on top of the last hidden layer:

$$f_{NN}(x)\;=\;h_{L-1}{\left(x\right)}^{T}W_{L-1}+\;\beta_{L},$$

The prediction error is evaluated using sum of squares *Error*(**W**, ***β***) = ∑_*t*_(*y*_***t***_ − *f*_*NN*_(***x***_***t***_))^2^ regularized by weight decay $\alpha \sum_{l\;=\;1}\sum_{i}W_{li}^{2}$.

To train the NN, we minimized the regularized prediction error using RMSprop [69] with momentum 0.9. Only structures with equal width in each layer were considered in this paper. The final network was selected by cross validation among the networks trained with the following hyper-parameter settings: depth of 1–4; width of 100, 200, 300, 400, 500; weight-decay parameter *α* ranging over 0.001, 0.005, 0.01, 0.05, 0.1, 0.5; and the number of training epochs of 100, 200, 300, 400, 500. The final network had depth of 3, width of 200, weight-decay parameter of 0.05, and was trained over 100 epochs.

Next, we estimated the prediction error associated with our NN with a leave-SynCom-out cross-validation experiment; in this scheme, the model was trained on all but one synthetic community and evaluated on that held-out synthetic community. We calculated the mean Pi content prediction error on each held-out synthetic community as the cross-validation error for a single fold. Based on the cross-validation experiment, we chose the best NN model architecture, which had 3 hidden layers and 200 neurons in each layer, as shown in (Fig 7A).

We note that there are more parameters than observations in the NN model. However, the number of parameters in a model is not a proper estimate of the complexity in the model. By choosing proper regularization parameters (early stopping, weight decay), the model can be trained without overfitting, even if it has more parameters than the number of observations [70]. The cross-validation error in Fig 7B and the validation error in Fig 7F both indicate that the model is not overfitting, because the model is able to generalize to never-seen-before synthetic communities.

Besides the leave-SynCom-out cross-validation experiment, we also performed a leave-biological-replicate-out cross-validation experiment. For this experiment, we used the data from 1 biological replicate to train each of the 3 models and used those trained models to predict the other biological replicate. The mean cross-validation error is shown in Table 5.

**Table 5:** Leave-biological-replicate-out cross-validation error.

Fold	LM	INT	NN
Training on BioRep 1 and predicting on BioRep 2	0.3097	0.2475	**0.2359**
Training on BioRep 2 and predicting on BioRep 1	0.3094	0.2636	**0.2472**

Table shows the mean cross-validation error associated with the leave-biological-replicate-out cross-validation experiment for the 3 models used: LM, INT, and NN. In bold is the cross-validation error for the NN model that has the lowest cross-validation error of all models used.

Abbreviations: BioRep, biological replicate; INT, linear model with interaction; LM, linear model; NN, neural network.

#### f. Sensitivity in different models

Neural network methods are often regarded as “black-box” methods. However, Neural networks are not totally opaque and uninterpretable. There are many recent studies aimed to reveal and extract information from Neural networks [71][72][73]. In order to extract biological insights from the NN, we performed a perturbation-based approach to quantify and understand the influence of specific features under different contexts.

As our inputs are all binary features, we define sensitivity as the difference in the output between the “on” (1) and “off” (0) state of a particular feature. The sensitivity of the prediction with respect to each input context is

$$\rho_{i}(x)\;=\;f(x_{\left(x_{i}\;=\;1\right)})-\;f(x_{\left(x_{i}\;=\;0\right)}),$$

where **x**_(xi = 1)_ means change the ith feature in **x** to 1 and keep other features fixed.

In a linear model, **ρ**_**i**_(**x**) = **w**_**i**_. Hence, the sensitivity in a linear model is independent of the input context. In a linear model with interaction features, we have

$$\rho_{i}\left(x\right)=w_{i}+\Sigma_{i\neq j}x_{j}\;{ϴ}_{i,j}.$$

Therefore, a linear model with interaction features has an input context–dependent sensitivity. In NNs, the exact form of **ρ**_**i**_(**x**) is complicated and hard to express in a closed form. In this paper, we calculate sensitivity **ρ**_**i**_(**x**) numerically. Fig 7C shows the sensitivity of different models. For context-specific models, we consider all possible inputs with synthetic community of size |**S**| = 2.

The code to estimate sensitivity of the different models is available at https://github.com/clingsz/wheelPi.

#### g. Generation of candidate block replacements

We sought to identify cases in which replacing (swapping) one of the bacterial blocks (B) for a different block (C) in a reference synthetic community S_1_ = {A, B}, thereby producing a different synthetic community S_2_ = {A, C}, would produce a significant increase in shoot Pi content under a certain phosphate condition (pretreatment p phosphate-level q). We can use a trained model to estimate the mean and variance of the plant phenotypic output in every possible synthetic community. The expected Pi content for any input of interest can be calculated as **f**(**x**_b,p,q,S_). The worst-case variance estimate is used in our prediction, in which the largest residual variance (difference between observed value and predicted value) related to a bacterial block is transferred from the training data to all related conditions. Specifically,

$${\hat{z}}_{b,p,q,S,r}=\;f\left(x_{b,p,q,S}\right)+\;z_{b,p,q,M,r}\;-\;f\left(x_{b,p,q,M}\right)$$

$$M=\left\{e_{1}^{*},e_{2}^{*}\right\}=\;\underset{e_{1}\in S\;or\;e_{2\;}\in S}{\text{argmax}}v_{b,p,q,\left\{e_{1},e_{2}\right\}}$$

Six predicted samples are generated for every design:

$${\hat{z}}_{p,q,S}=\{{\hat{z}}_{b,p,q,S,r}\;|\;b\;\in \left\{1,2\right\}\;\wedge r\;\in \left\{1,2,3\right\}\;\}$$

Given any 2 synthetic communities S_1_, S_2_, under a certain phosphate condition, p and q, the mean difference is $\frac{1}{6}\sum_{b\;=\;1}^{2}\sum_{r\;=\;1}^{3}{\hat{z}}_{b,p,q,S_{2},r}\;-\;{\hat{z}}_{b,p,q,S_{1},r}$ and the *p*-value is calculated from a 2-sample *t* test on ${\hat{z}}_{p,q,S_{2}}$ and ${\hat{z}}_{p,q,S_{1}}$.

The code to generate candidate block replacements is available at https://github.com/clingsz/wheelPi.

## Supporting information

S1 Fig Primary metabolite profiles differ in root exudates from plants grown in different Pi regimens for 24 hours. (A) Schematic representation of the pipeline used for screening the bacteria collection using plant exudates. For root exudate preparation, Col-0 seeds were germinated on Johnson medium, 0.5% sucrose, supplemented or not with 1 mM Pi. After 7 days of growth, seedlings were transferred to the opposite concentration of Pi from the solid growth conditions (i.e., plants that were initially grown in 1 mM Pi were transferred to liquid medium with no supplementation of Pi [exudates +/−] and vice versa [exudates −/+]). Plants were grown in liquid media with agitation (30 rpm) for 24 hours in a growth chamber in a 16-hour light/8-hour dark regime (24°C/21°C). (B) For screening of the bacteria isolate collection in different plant root exudates, a single bacterial colony was inoculated in 200 μL of 2×YT medium in a 96-well polystyrene plate. Bacterial cultures were grown for 24 hours, and then all cultures were diluted 1/10 into the different plant exudates or into control media alone and grown for another 3 days. Exudate and media samples without bacteria were included as controls of contamination. The optical density at 600 nm was measured every 3 hours during the day and every 14 hours during the night. (C) Heat map showing the standardized abundances of all primary metabolites identified in exudate and control media with 1 mM Pi (control +) or without Pi (Control −) samples (S1 Table and Materials and methods 1e). (D) Heat map showing the log(fold-change) of the 76 metabolites identified in the exudates, with respect to the media alone (Materials and methods 1e). In both cases, plants were grown in the conditions defined in (A). As additional controls, we transferred plants germinated without Pi to liquid Johnson medium not supplemented with Pi (exudates −/−) and plants germinated with 1 mM Pi to Johnson medium supplemented with 1 mM Pi (exudates +/+). We also analyzed samples of liquid Johnson medium supplemented with 1 mM Pi (control +) and without Pi supplementation (control −), each in the absence of bacteria. Col-0, Columbia-0; Pi, phosphate; rpm, revolutions per minute.
(TIF)

S2 Fig Bacteria differentially respond to root exudates. (A) Upper: 16S rRNA based phylogenetic-tree bacterial strains. Lower: Heat map showing the z-scores for bacterial in vitro growth features. Feature names for rows at the right of the figure are the following: −Pi (about 5 μM Pi) and +Pi (1 mM Pi) are media with no exudates; −Pi_+P (exudates generated from plants germinated on −Pi and transferred to +Pi media); +Pi_−P (exudates generated from plants germinated in +Pi and transferred to −Pi media) (S1A Fig and Materials and methods 1b). For the rest of the rows, the prefix indicates the condition; the suffix indicates the measurement. Left: *p*-values (log_10_) from Pagel’s *λ* test for phylogenetic signal. Features were normalized by row. Only 395/440 strains that were both included in the in vitro assays and had a high-quality full-length 16S are included. (B) Groups of bacteria with differential responses to Pi and root exudates. Heat map shows z-scores of the AUC measurements for the in vitro bacterial growth curve for growth in various media. Rows correspond to the features with the same labels as in part (A). The AUC has been standardized by column, which corresponds to each of the strains (*n* = 440) tested after quality control (including those that had no high-quality full-length 16S). The top 2 panels are control conditions, as defined in (A). The bottom 2 conditions are media with exudates collected from plants, as defined in (A). Strains were grouped (1–10) by hierarchical clustering using the euclidean distance and the complete linkage method. AUC, area under the curve; HMT, mean time to reach half maximum density; L3M, mean density over last 3 measurements; M, minusP; MAX, maximum density; MGS, maximum growth rate; MLF, log_2_(PM/M); MP, minus2PlusP; P, plusP; Pi, phosphate; PLF, log_2_(MP/P); PM, plus2MinusP; 16S, small subunit ribosomal rRNA gene.
(TIF)

S3 Fig Activation of the phosphate starvation response modulates the outcome of binary bacteria–plant interactions. (A) Colonization of the different plant organs and surrounding agar by 6 bacterial strains. Bacterial strains were selected according to their performance in binary-association assays: 3 strains increased (green arrows) and another 3 decreased (red arrows) the shoot Pi content (see panel E). Data points are colored by bacterial strain. Letters at the top of each panel denote statistical significance of Tukey post hoc analysis of an LM. In all cases, plant tissue and agar samples were crushed, serially diluted, and plated and c.f.u per gram of original material was determined. (B) Shoot Pi content normalized by the shoot fresh weight in mg of seedlings germinated on Johnson media, 0.5% sucrose, supplemented with 1 mM Pi (+Pi), not supplemented with Pi (−Pi), or supplemented with 1 mM Phi (+Phi). Numerical values that underlie the data displayed in the panels are in https://github.com/surh/wheelP. (C) Expression analysis of the reporter construct *IPS1*:*GUS* in plants germinated in the conditions described in (B). In this construct, the *IPS1* promoter, highly induced by low Pi, controls the expression of GUS. (D) Distribution of shoot Pi content normalized by the shoot fresh weight in mg in plants cocultured with individual bacterial strains (+Bacteria) or in axenic condition (No Bacteria) across a 3 × 2 combination of 3 conditions used for plant germination (1 mM Pi, about 5 μM Pi, and 1 mM phosphite), and 2 Pi concentrations (30 μM Pi and 100 μM Pi) that were applied concomitant with each bacterial strain. (E) Table shows the fold change in shoot Pi accumulation between plants inoculated with an individual bacterial strain, listed at left, and plants grown axenically. Cells with a color block indicate statistically significant Pi concentration changes from axenic control (*q*-value < 0.05; ANOVA and Tukey test). c.f.u, colony-forming unit; *GUS*, beta-glucuronidase; *IPS1*, INDUCED BY PHOSPHATE STARVATION1; LM, linear model; Phi, phosphite; Pi, phosphate.
(TIF)

S4 Fig There is no correlation between bacterial performance in response to root exudates and the effect of individual isolates on shoot phosphate accumulation. Scatterplots showing correlation between the AUC for bacterial growth curves (x-axis) in different exudates (exudate −/+ and exudate +/−) and control (control − and control +) conditions and the mean log(fold-change) in Pi accumulation across all 4 conditions (−Pi_100 μM, −Pi_30 μM, +Pi_100 μM, +Pi_30 μM). Dots are color coded by their in vitro growth condition; the blue line shows the loess smoother and the grey shade the 95% confidence interval on the smoother. AUC, area under the curve; loess, local regression; Pi, phosphate.
(TIF)

S5 Fig Synthetic communities differentially modify plant phenotypes. Changes in plant phenotypes induced by synthetic communities, compared with axenically grown seedlings. In each plot, the 4 quadrants represent the 4 media conditions tested, with pretreatment as rows (labels at right) and posttreatment as columns (labels at top). X- and y-axes show the 9 bacterial blocks (see S4 Table for strains in each block). The lower triangle cells in each panel show the phenotype change induced by a synthetic community composed of the 2 blocks indicated by its x- and y-coordinates. In all plots, 0 (bordered box with white interior) represents no change in the corresponding phenotype with respect to axenically grown plants, and the color scale indicates more (green) or less (magenta) than axenically grown plants. Statistically significant differences (*p*-value < 0.05) are indicated with an “x” inside each square. The phenotypes analyzed are: shoot Pi accumulation (Pi content), primary root elongation (Main root elongation), shoot area, and total root network. The values for Pi content and shoot area indicate log(fold-change) with respect to axenically grown plants. The values for main root elongation and total root network represent difference with respect to axenically grown plants. Pi, phosphate.
(TIF)

S6 Fig Analysis of bacterial colonization in the synthetic community experiments. (A) Constrained multidimensional scaling, in which bacterial relative abundances were conditioned on sequencing depth, biological replicate, and sequencing batch. The remaining unexplained variance was then subjected to MDS based on the Bray-Curtis dissimilarity. (B) Bacterial taxonomic distributions of synthetic communities following colonization (see S4 Table for strains in each block). Top panel shows the theoretical input for each of the 14 original synthetic communities based on the 16S rRNA sequences of the bacterial strains used in the 2 blocks that comprise each synthetic community. Bottom panels show agar (left) and root (right) bacterial taxonomic distributions for each of the 14 synthetic communities. For each synthetic community, all individual sequenced samples from agar or roots with at least 400 reads are shown, and samples are sorted by biological replicate within each synthetic community. For some synthetic communities, there were no samples that passed the minimum read threshold, and they are not shown. Colors indicate the proportion of reads that were mapped to strains belonging to the corresponding bacterial order. The phosphate conditions in the media are indicated on the right side (Pi treatments): the axenic germination phosphate condition is indicated first and then the Pi concentration applied concomitant with bacteria. (C) Panels show agar (left) and root (right) bacterial block contributions to colonization for each of the 14 synthetic communities. For each synthetic community, all individual samples with at least 400 reads are shown, and samples are sorted by biological replicate within each community. For some synthetic communities, there were no samples that passed the minimum read threshold, and they are not shown. Colors indicate the proportion of reads that were mapped to strains belonging to the corresponding bacterial block, as defined in Fig 3B. The theoretical input for each block would be 50%. The phosphate conditions in the media are indicated on the right side (Pi treatments), as in panel B. Numerical values that underlie the data displayed in the panels are in https://github.com/surh/wheelP. MDS, multidimensional scaling; Pi, phosphate; 16S, small subunit ribosomal rRNA gene.
(TIF)

S7 Fig Bacterial relative abundance provides no information regarding host phenotypic output. Comparisons between measured changes (x-axis) in plant phenotypes caused by synthetic communities, with respect to axenically grown plants, and expected changes (y-axis) from models that use bacterial relative abundance (red) or only bacterial presence/absence (cyan). In each plot, the 4 panels represent the 4 media conditions tested, with germination conditions as rows and Pi treatment as columns. Each point represents a synthetic community (*n* = 14); the x-axis corresponds to the color scale in Fig 3C and the y-axis shows the results from additive models that either consider (red) or ignore (cyan) relative abundances. The standard error from both the measured and estimated change is shown for each point. The lines represent the least squares regression on the points from each panel, and the grey shade indicates the 95% confidence interval on the regression lines. The *R*^*2*^ is shown on each panel and for each model. For all axes, 0 represents no change with respect to axenically grown plants. The values for Pi content and shoot area are indicated as log (fold-change) with respect to axenically grown plants. The values for primary root elongation and total root network represent the difference with respect to axenically grown plants. In all cases (16/16), the *R*^*2*^ for a model that incorporates relative abundance information was smaller than the model that ignored it (*p*-value = 0.000381, Wilcoxon signed-rank test). Numerical values that underlie the data displayed in the panels are in https://github.com/surh/wheelP. Pi, phosphate; *R*^*2*^, coefficient of determination.
(TIF)

S8 Fig Activation of the transcriptional phosphate starvation response as a function of shoot Pi content. In the figure, each point represents a bacterial synthetic community. The x-axis gives the average of shoot Pi content and the y-axis the average of the phosphate starvation response marker genes activation. Data from 2 independent biological replicates are shown, and a loess smoother is shown as a blue line. The grey zone represents the 95% confidence interval of the smoother. Each panel represents a condition. Only conditions that ended with 30 μM Pi are shown, because no activation was observed in the other conditions. loess, local regression; Pi, phosphate.
(TIF)

S9 Fig Plants growing at low Pi with synthetic communities generally induced phosphate starvation response genes and modified the expression of immune system–related genes. (A) Hierarchical clustering of the approximately 17,000 most variably expressed genes in our RNA-Seq experiment (S6 Table). Rows represent the average from all samples with a given bacterial treatment in each condition, and columns represent genes. Genes are clustered according to their expression profiles. (B) Gene ontology enrichments for clusters c1, c2, and c4 defined in (A). Gene sets (c1, c2) matched the expression pattern of the phosphate starvation response marker genes. Gene ontology enrichment analysis revealed that these clusters are enriched in defense genes (S7 Table). Cluster c1 also includes stress response genes, like low Pi responsive genes (S7 Table). Cluster c2 showed an overrepresentation of salicylic acid signaling genes and other genes associated with plant immunity (S7 Table). Cluster c3 contained numerous genes related to the metabolism of membrane phospholipids. These genes are more induced in 30 μM Pi and are potentially involved in the metabolic replacement of phospholipids by sulfolipids in Pi-deficient plants (S7 Table). We found in cluster c4 enrichment in plant immune system function, specifically in JA response. (C) Differentially expressed genes in response to bacteria are mainly associated with PTI and SA (S7 Table). The first column shows the log(fold-change) in the expression of 1,238 genes that are differentially expressed between plants that encountered bacteria versus axenically grown plants (FDR < 0.01). Positive values (green) correspond to genes more expressed in plants that encountered bacteria, while negative values (magenta) correspond to genes more highly expressed in axenically grown plants. The following columns indicate genes annotated as related to defense, SA, JA, or ABA, according to gene ontologies. The numbers on top indicate the number of genes in each functional class, and the asterisks indicate functions that are enriched among more highly expressed genes in the presence of bacteria (green) or in axenically grown plants (magenta). ABA, abscisic acid; c1–c4, clusters 1–4; FDR, false discovery rate; JA, jasmonic acid; PAMP/MAMP, Pathogen- or microbe-associated molecular pattern; Pi, phosphate; PTI, PAMP/MAMP-triggered immunity; RNA-seq, RNA sequencing; SA, salicylic acid.
(TIF)

S10 Fig Summary of GO enrichment analysis for differentially expressed genes in response to bacterial blocks. The left panel shows GO terms (**GO names,** y-axis) enrichments among down-regulated genes (**down**), and the right panel GO terms enrichments among up-regulated genes (**up**). The x-axis indicates the bacterial block for the corresponding enrichment test. Color indicates the statistical significance after controlling for multiple testing (−log10(FDR)). Nonsignificant enrichments (FDR ≥ 0.05) are shown in white. A significant result means that a particular GO term is enriched among the genes that are down-or up-regulated in response to specific bacterial blocks. Enrichment analysis was performed on differentially expressed genes from S6 Table. FDR, false discovery rate; GO, gene ontology.
(TIF)

S11 Fig Shoot Pi content has the strongest signal-to-noise ratio of all plant phenotypes tested. The bar plot shows the signal variance and noise variance for all plant phenotypes tested: shoot Pi content (Pi), Primary root elongation (Main), shoot area (Area), and total root network (Net). See also Materials and methods 4b. Numerical values that underlie the data displayed in the panel are in https://github.com/surh/wheelP. Pi, phosphate.
(TIF)

S12 Fig Analysis of the bacterial colonization in the neural network validation experiment. Taxonomic (left) and block (right) bacterial abundances for inoculum (top), agar (middle), and root (bottom) samples from the 20 synthetic communities of the validation experiment (see S4 Table for strains in each block). For each synthetic community, all individual sequenced samples with at least 400 reads are shown. For some synthetic communities, there were no samples that passed the minimum read threshold, and they are not shown. Colors indicate the proportion of bacterial reads that were mapped to the corresponding bacterial order (left) or block (right). All plants in this experiment were axenically germinated in a medium without phosphate supplementation (−Pi) and then transferred to 30 μM Pi, concomitant with addition of the different bacterial synthetic communities. Numerical values that underlie the data displayed in the panels are in https://github.com/surh/wheelP. Pi, phosphate.
(TIF)

S1 Table List of primary metabolites identified in plant exudates. The table shows the primary metabolite profiles from root exudates and control media samples obtained using ALEX-CIS GCTOF MS at NIH West Coast Metabolomics Center (University of California, Davis, CA). In the table, **BinBase name** denotes the name of the metabolite if the peak has been identified. The retention index column (**ret.index**) details the target retention index in the BinBase database system. **quant mz** details the m/z value that was used to quantify the peak height of a BinBase entry. The column **BB id** denotes the unique identifier for the GCTOFMS platform. It is given for both identified and unidentified metabolites in the same manner. The **mass spec** column details the complete mass spectrum of the metabolite given as mz: intensity values, separated by spaces. The **KEGG** identifier gives the unique identifier associated with an identified metabolite in the community database KEGG LIGAND DB. The **PubChem** column denotes the unique identifier of a metabolite in the PubChem database. The **InChI key** identifier gives the unique chemical identifier defined by the IUPAC and NIST consortia. The actual data are given as peak heights for the quantification ion (mz value) at the specific retention index. Raw data were normalized according to NIH West Coast Metabolomics Center (University of California, Davis, CA) quality standards. Plants were germinated on Johnson medium plus 0.5% sucrose, supplemented or not with 1 mM Pi, in a horizontal position. After 7 days of growth, seedlings were transferred to a 12-well plate containing 3 mL of liquid Johnson medium per well. We transferred the seedlings to the opposite concentration of Pi from the solid growth conditions (i.e., plants that were initially grown in 1 mM Pi were transferred to liquid medium with no supplementation of Pi [exudates +/−] and vice versa [exudates −/+]). As controls, we transferred plants germinated without Pi to liquid Johnson medium not supplemented with Pi (exudates −/−) and plants germinated with 1 mM Pi to Johnson medium supplemented with 1 mM Pi (exudates +/+). We also analyzed samples of liquid Johnson medium supplemented with 1 mM Pi (control +) and without Pi supplementation (control −). Pi, phosphate.
(XLSX)

S2 Table Bacterial in vitro growth curves. This table provides the data underlying S2 Fig. Four hundred forty bacterial strains were individually tested for their ability to grow under different Pi concentrations and plant exudates from different Pi starvation conditions. The first column (Strain) provides the strain ID for all tested strains. Column Group indicates the group to which a given strain belongs, as represented in S2B Fig, and column r.squared is a goodness-of-fit measurement that indicates how well a strain follows the group behavior. Groups were defined by hierarchical clustering of the area under the curve (time versus OD). The next block of columns (Growth parameters) contains the values of all parameters calculated for each growth curve. These data are also on the “Features” data object provided in the github repository. See legend on S2A Fig for the meaning of abbreviations. Taxonomy of each strain that had an available 16S sequence is provided as reported by RDP (*n* = 395). Kingdom, phylum, class, order, family, and genus are given. Only 395 strains had a high-quality full-length 16S gene sequence and could be annotated. Finally, the reference sequence for the 395 strains that had it is provided. Raw data underlying these results are provided in the ‘All.filtered’ data object of the associated github repository. ID, identification; OD, optical density; Pi, phosphate; RDP, Ribosomal Database Project; 16S, small subunit ribosomal rRNA gene.
(XLSX)

S3 Table Plant–bacterium binary-association assays. This table provides the data underlying Figs 2B, 2C and 3A. The first group of columns shows the **Log(fold-change in shoot Pi content)** caused by each individual strain on *Arabidopsis thaliana* in each of 4 Pi starvation conditions. The **Mean** log(fold-change) across conditions is also provided, and this value was used to sort the strains within each functional class in Fig 3A. The **Standard errors** of the log(fold-change) and the *p*-values corrected for multiple testing (***q*-values**, Benjamini-Hochberg method) are also provided. The functional class (**Functional.class**) and the specific **Block** to which each strain belongs are given at the end. These assignments are also indicated in Fig 3A. This table is also provided as the “binP” data object in the associated github repository. The raw data that underlie the presented statistics are also provided as data object “binP.all.” Pi, phosphate.
(XLSX)

S4 Table List of strains used in the synthetic communities experiments. For each of the strains used in the synthetic communities experiments, we indicate its ID, its taxonomy, the blocks of bacteria to which it belongs, the ID in the binary-association assays, the taxon_oid, which can be used to retrieve their full genome from the DOE JGI IMG database, and the full strain name. The information used in this table and additional data can be found in https://github.com/surh/wheelP under the descriptor “Tax. Colonization.” DOE, Department of Energy; ID, strain identifier; IMG, Integrated Microbial Genomes; JGI, Joint Genome Institute.
(XLSX)

S5 Table Table shows the effect of bacterial consortia on shoot Pi accumulation. Tab (a) shows the effect of individual bacterial blocks (**Block effects**) and tab (b) the effect of synthetic communities (**SynCom effects**) on shoot Pi accumulation. For each bacterial block (a) or synthetic community (b), its effect on shoot Pi accumulation was determined via ANOVA (Materials and methods 3j). For each test, we indicate the starting (**StartP**) and final (**EndP**) Pi concentrations, the estimated log(fold-change) in Pi content with respect to no bacteria (**Estimate**), the standard error of that estimate (**SE**), the associated *t* statistic (**t.value**), and the *p*-value of a 2-sided test (**p.value**). Pi, phosphate; SynCom, synthetic community.
(XLSX)

S6 Table Table contains the information about the differential expression tests for the 20,494 genes analyzed in the RNA-Seq experiment. For each comparison, we provide the log2(fold-change) in relative expression between the 2 groups being compared (**logFC**), the log2 average counts per million (**logcPM**), the *p*-value of the test (**Pvalue**), and the *p*-value adjusted for multiple testing (**FDR**). We performed the following comparisons: comparison of samples that were inoculated with bacteria versus the axenic controls (**Bacteria vs No Bacteria**), comparison of the combined effect of positive blocks versus the combined effect of negative blocks (**Positive vs Negative**), comparison of the combined effect of negative blocks among both pretreatments in the 30 μM posttreatment (**(+Pi vs −Pi) | 30 μM**), comparison of blocks N2 and N3 in the 30 μM posttreatment (**(N2 vs N3) | 30 μM**), comparison of blocks N1 and N3 in the 30 μM posttreatment (**(N1 vs N3) | 30 μM**), comparison of blocks N1 and N2 in the 30 μM posttreatment (**(N1 vs N2) | 30 μM**), and the main effect of each block (**P1, P2, P3, I1, I2, I3, N1, N2, N3**). See Materials and methods and code for details on how comparisons were made. FDR, false discovery rate; logFC, log Fold Change; RNA-Seq, RNA sequencing.
(XLS)

S7 Table GO enrichment analysis of genes differentially expressed in *Arabidopsis thaliana* Col-0 in response to different bacterial synthetic communities. In the Table, tab (a) (**Cluster assignments**) contains the 17,106 most variably expressed genes in *A*. *thaliana* seedlings in our RNA-Seq experiment in response to different bacterial synthetic communities. Genes are clustered according to their relative expression profiles from all samples with a given bacterial treatment in each condition. The table indicates the cluster assignment for each gene. Tabs (b)–(h) show the GO enrichment analysis for each cluster. Analysis was performed in the PlantGSEA web platform. Each tab contains the following information: descriptive name of the GO category (**Description**), ID in the GO database (**GO ID**), the total number of *A*. *thaliana* genes annotated with the corresponding GO term (**Total**), the number of *A*. *thaliana* genes in the corresponding cluster annotated with the corresponding GO term (**Found**), *p*-value of the enrichment test (***p*-value**), and the adjusted *p*-value for multiple testing, using the Benjamini-Hochberg method (***q*-value**). See also S9A Fig, RNA-Seq clustering. Col-0, Columbia-0; GO, gene ontology; GO ID, GO identifier; RNA-Seq, RNA sequencing.
(XLSX)

S8 Table List of the top 25 hypotheses generated by the neural network. The table contains the 25 most significant hypotheses generated by our neural network for plants germinated without Pi supplementation and transferred to 30 μM Pi in the presence of the synthetic communities. The "Reference" column shows the original synthetic communities, into which we switched a bacterial block to generate "New" synthetic communities that were predicted to increase significantly the Pi content in the plant shoot. The predicted increase in Pi content is shown in the "Predicted Mean Difference" column, and the log10 of the *p*-value associated with the prediction is shown in "log10 Predicted *p*-value" column. Pi, phosphate.
(XLSX)

S9 Table Validation results of the top 25 hypotheses generated by the neural network. The table contains the validation results for the 25 most significant hypotheses generated by our neural network for plants germinated without Pi supplementation and transferred to 30 μM Pi with the synthetic communities. The "Reference" column shows the original synthetic communities into which we switched a bacterial block to generate "New" synthetic communities that were predicted to significantly increase the Pi content in the plant shoot. The actual increase in mean Pi content from reference to previously untested synthetic communities is shown in the "Mean Difference" column, and the log10 of the *p*-value is shown in "log10 *p*-value" column. Color represents the experimental result: significant increase (green), nonsignificant increase (yellow), nonsignificant decrease (pink), and significant decrease (red). Pi, phosphate.
(XLSX)

## Abbreviations

AUC: area under the curve
HMT: mean time to reach half maximum
INT: linear model with pairwise interaction
*IPS1*: INDUCED BY PHOSPHATE STARVATION1
L3M: mean density over the last 3 measurements
leakyReLU: leaky Rectified Linear Unit
LM: linear model
MAX: maximum optical density
MGS: maximum growth rate
NN: neural network
OD: optical density
Phi: phosphite
*PHO1*: PHOSPHATE1
*PHO2*: PHOSPHATE2
PHR1: PHOSPHATE STARVATION RESPONSE1
Pi: phosphate
RPKM: reads per kilobase per million
SNR: signal-to-noise ratio
SynCom: synthetic community
