# Supplementary figures and images for: Design of synthetic bacterial communities for predictable plant phenotypes
Source: PLoS Biol. 2018 Feb 20;16(2):e2003962. doi: 10.1371/journal.pbio.2003962 (PMC5819758; doi:10.1371/journal.pbio.2003962)

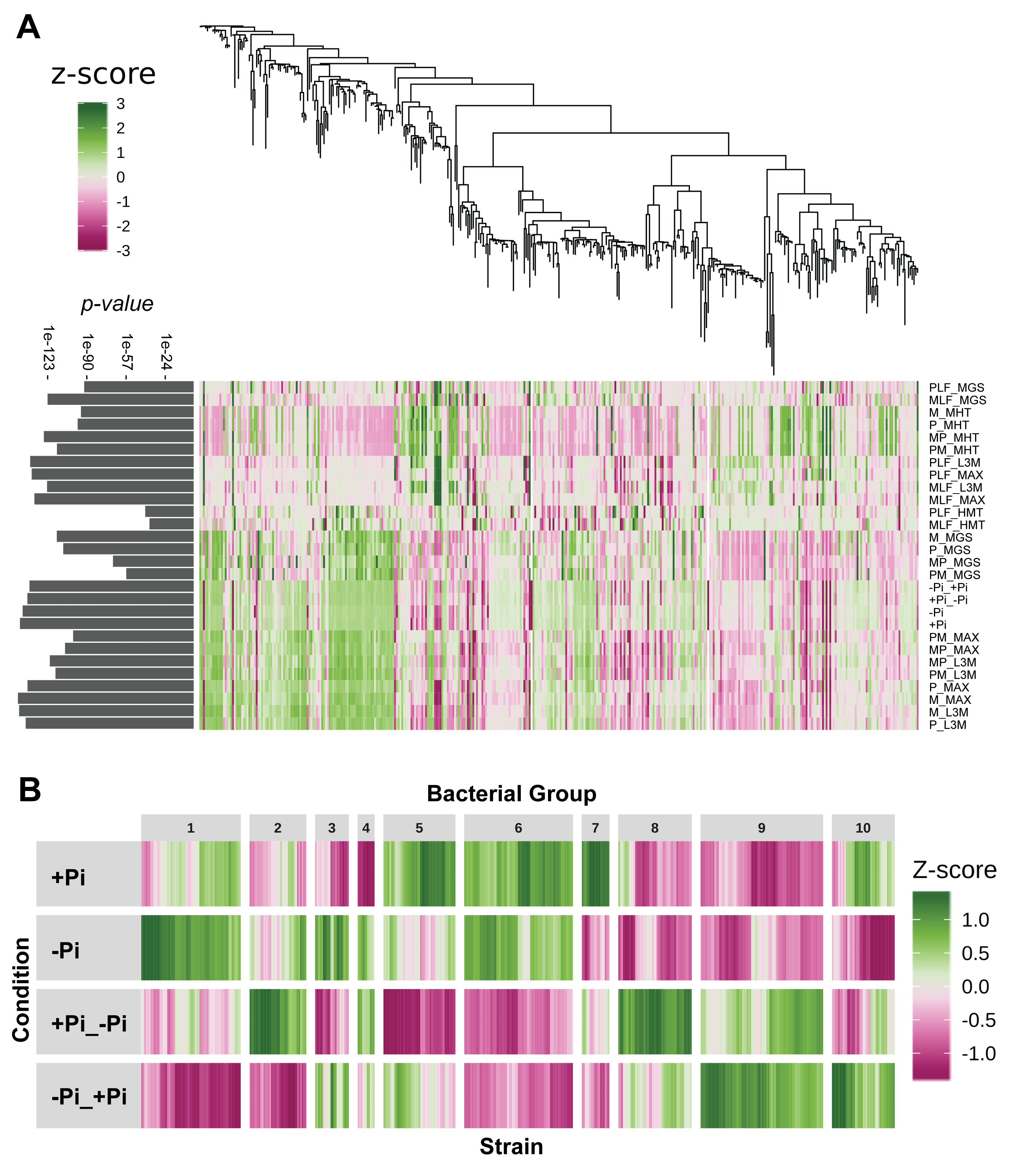

Supplement: S2 Fig — (A) Upper: 16S rRNA based phylogenetic-tree bacterial strains. Lower: Heat map showing the z-scores for bacterial in vitro growth features. Feature names for rows at the right of the figure are the following: −Pi (about 5 μM Pi) and +Pi (1 mM Pi) are media with no exudates; −Pi_+P (exudates generated from plants germinated on −Pi and transferred to +Pi media); +Pi_−P (exudates generated from plants germinated in +Pi and transferred to −Pi media) (S1A Fig and Materials and methods 1b). For the rest of the rows, the prefix indicates the condition; the suffix indicates the measurement. Left: p-values (log10) from Pagel’s λ test for phylogenetic signal. Features were normalized by row. Only 395/440 strains that were both included in the in vitro assays and had a high-quality full-length 16S are included. (B) Groups of bacteria with differential responses to Pi and root exudates. Heat map shows z-scores of the AUC measurements for the in vitro bacterial growth curve for growth in various media. Rows correspond to the features with the same labels as in part (A). The AUC has been standardized by column, which corresponds to each of the strains (n = 440) tested after quality control (including those that had no high-quality full-length 16S). The top 2 panels are control conditions, as defined in (A). The bottom 2 conditions are media with exudates collected from plants, as defined in (A). Strains were grouped (1–10) by hierarchical clustering using the euclidean distance and the complete linkage method. AUC, area under the curve; HMT, mean time to reach half maximum density; L3M, mean density over last 3 measurements; M, minusP; MAX, maximum density; MGS, maximum growth rate; MLF, log2(PM/M); MP, minus2PlusP; P, plusP; Pi, phosphate; PLF, log2(MP/P); PM, plus2MinusP; 16S, small subunit ribosomal rRNA gene. (TIF) [file pbio.2003962.s002.tif]

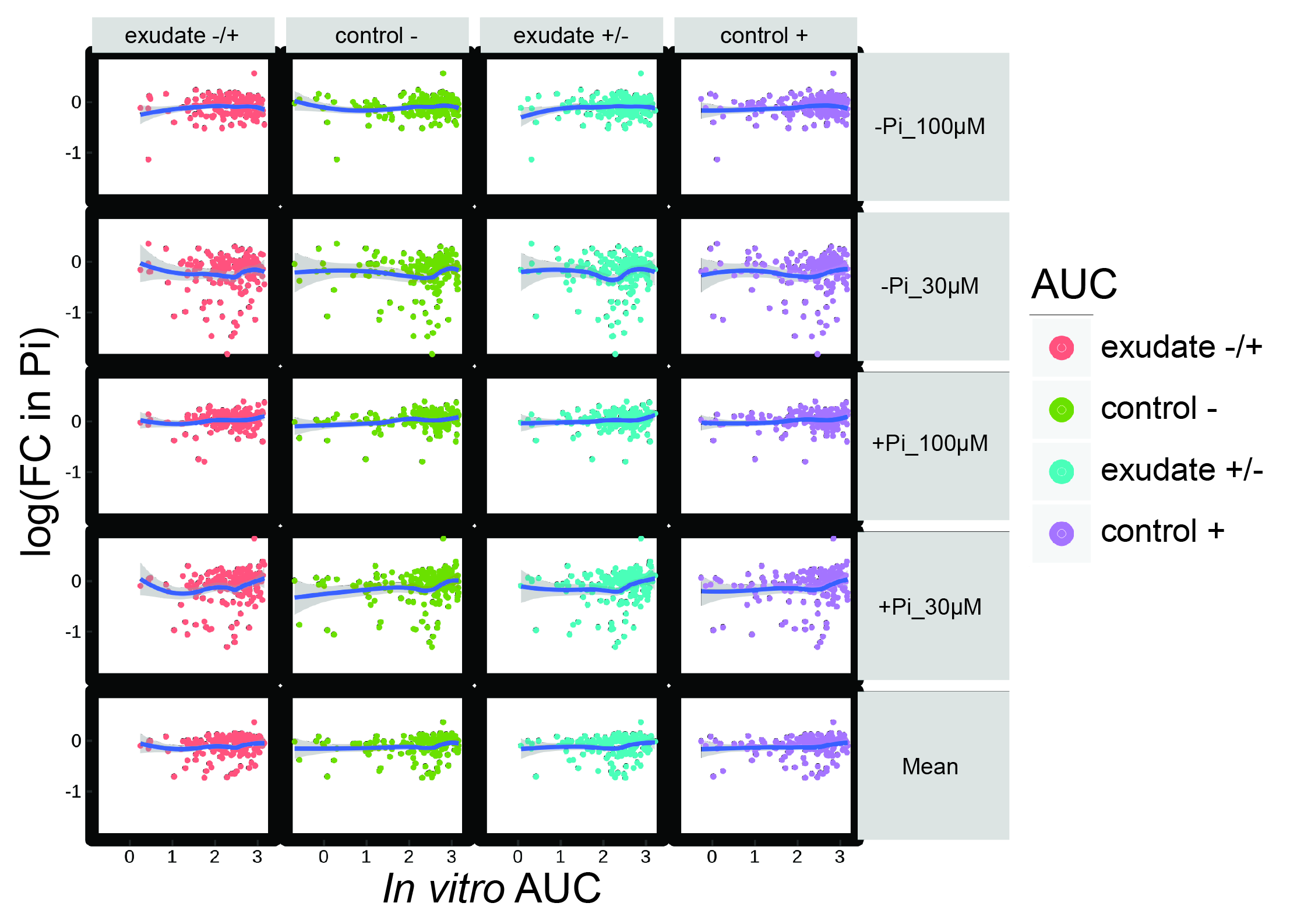

Supplement: S4 Fig — Scatterplots showing correlation between the AUC for bacterial growth curves (x-axis) in different exudates (exudate −/+ and exudate +/−) and control (control − and control +) conditions and the mean log(fold-change) in Pi accumulation across all 4 conditions (−Pi_100 μM, −Pi_30 μM, +Pi_100 μM, +Pi_30 μM). Dots are color coded by their in vitro growth condition; the blue line shows the loess smoother and the grey shade the 95% confidence interval on the smoother. AUC, area under the curve; loess, local regression; Pi, phosphate. (TIF) [file pbio.2003962.s004.tif]

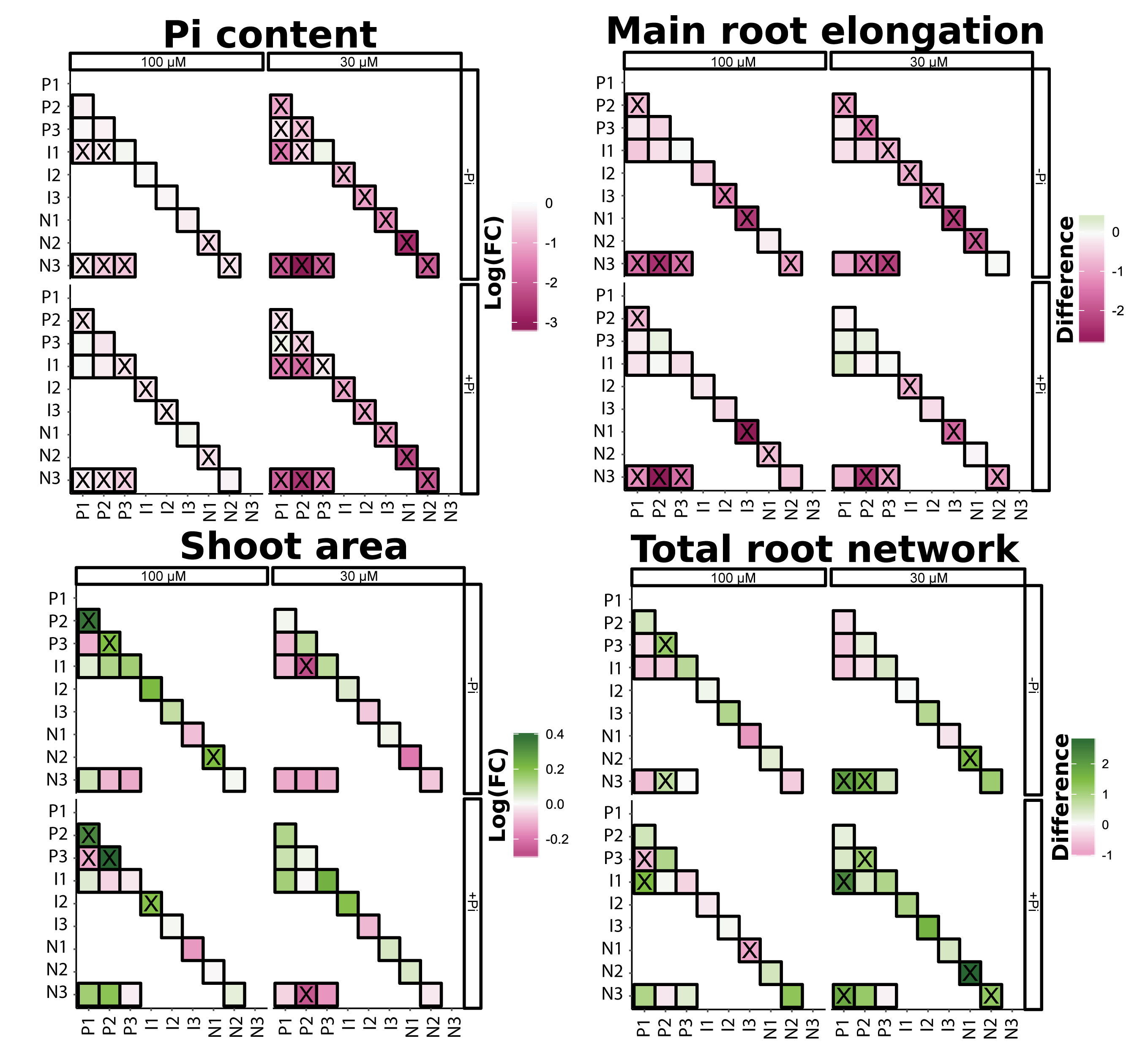

Supplement: S5 Fig — Changes in plant phenotypes induced by synthetic communities, compared with axenically grown seedlings. In each plot, the 4 quadrants represent the 4 media conditions tested, with pretreatment as rows (labels at right) and posttreatment as columns (labels at top). X- and y-axes show the 9 bacterial blocks (see S4 Table for strains in each block). The lower triangle cells in each panel show the phenotype change induced by a synthetic community composed of the 2 blocks indicated by its x- and y-coordinates. In all plots, 0 (bordered box with white interior) represents no change in the corresponding phenotype with respect to axenically grown plants, and the color scale indicates more (green) or less (magenta) than axenically grown plants. Statistically significant differences (p-value < 0.05) are indicated with an “x” inside each square. The phenotypes analyzed are: shoot Pi accumulation (Pi content), primary root elongation (Main root elongation), shoot area, and total root network. The values for Pi content and shoot area indicate log(fold-change) with respect to axenically grown plants. The values for main root elongation and total root network represent difference with respect to axenically grown plants. Pi, phosphate. (TIF) [file pbio.2003962.s005.tif]

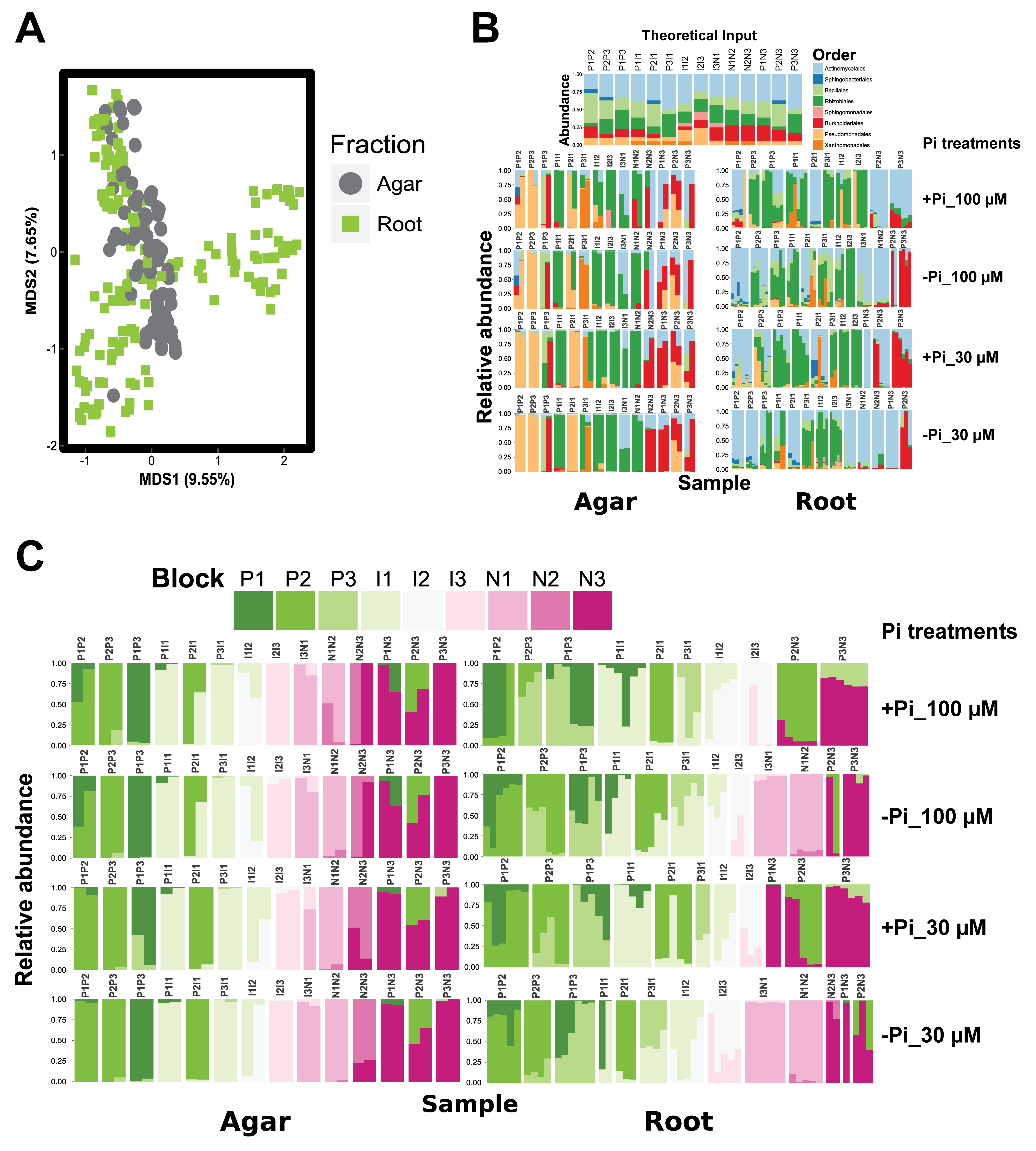

Supplement: S6 Fig — (A) Constrained multidimensional scaling, in which bacterial relative abundances were conditioned on sequencing depth, biological replicate, and sequencing batch. The remaining unexplained variance was then subjected to MDS based on the Bray-Curtis dissimilarity. (B) Bacterial taxonomic distributions of synthetic communities following colonization (see S4 Table for strains in each block). Top panel shows the theoretical input for each of the 14 original synthetic communities based on the 16S rRNA sequences of the bacterial strains used in the 2 blocks that comprise each synthetic community. Bottom panels show agar (left) and root (right) bacterial taxonomic distributions for each of the 14 synthetic communities. For each synthetic community, all individual sequenced samples from agar or roots with at least 400 reads are shown, and samples are sorted by biological replicate within each synthetic community. For some synthetic communities, there were no samples that passed the minimum read threshold, and they are not shown. Colors indicate the proportion of reads that were mapped to strains belonging to the corresponding bacterial order. The phosphate conditions in the media are indicated on the right side (Pi treatments): the axenic germination phosphate condition is indicated first and then the Pi concentration applied concomitant with bacteria. (C) Panels show agar (left) and root (right) bacterial block contributions to colonization for each of the 14 synthetic communities. For each synthetic community, all individual samples with at least 400 reads are shown, and samples are sorted by biological replicate within each community. For some synthetic communities, there were no samples that passed the minimum read threshold, and they are not shown. Colors indicate the proportion of reads that were mapped to strains belonging to the corresponding bacterial block, as defined in Fig 3B. The theoretical input for each block would be 50%. The phosphate conditions in the med [file pbio.2003962.s006.tif]

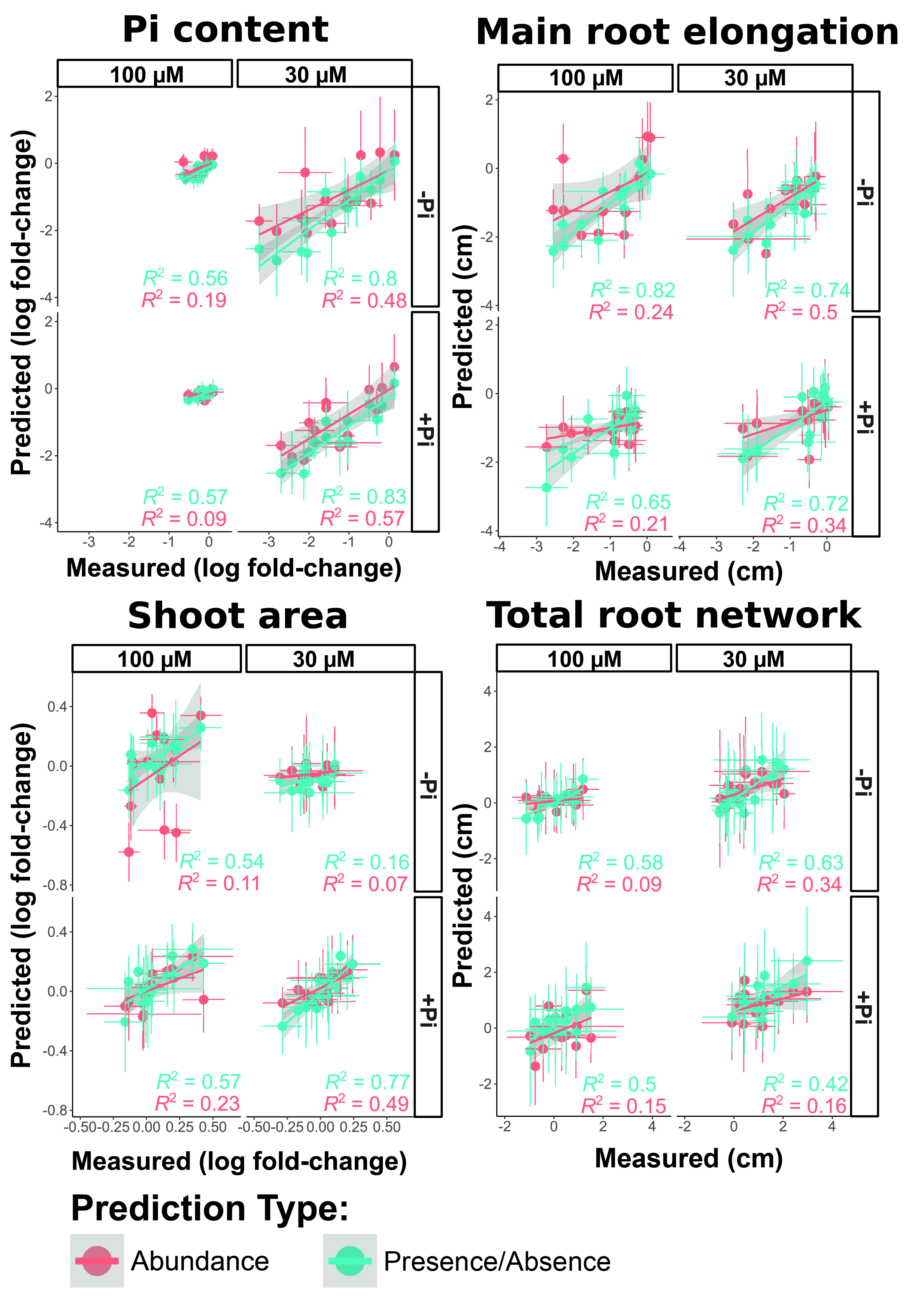

Supplement: S7 Fig — Comparisons between measured changes (x-axis) in plant phenotypes caused by synthetic communities, with respect to axenically grown plants, and expected changes (y-axis) from models that use bacterial relative abundance (red) or only bacterial presence/absence (cyan). In each plot, the 4 panels represent the 4 media conditions tested, with germination conditions as rows and Pi treatment as columns. Each point represents a synthetic community (n = 14); the x-axis corresponds to the color scale in Fig 3C and the y-axis shows the results from additive models that either consider (red) or ignore (cyan) relative abundances. The standard error from both the measured and estimated change is shown for each point. The lines represent the least squares regression on the points from each panel, and the grey shade indicates the 95% confidence interval on the regression lines. The R2 is shown on each panel and for each model. For all axes, 0 represents no change with respect to axenically grown plants. The values for Pi content and shoot area are indicated as log (fold-change) with respect to axenically grown plants. The values for primary root elongation and total root network represent the difference with respect to axenically grown plants. In all cases (16/16), the R2 for a model that incorporates relative abundance information was smaller than the model that ignored it (p-value = 0.000381, Wilcoxon signed-rank test). Numerical values that underlie the data displayed in the panels are in https://github.com/surh/wheelP. Pi, phosphate; R2, coefficient of determination. (TIF) [file pbio.2003962.s007.tif]

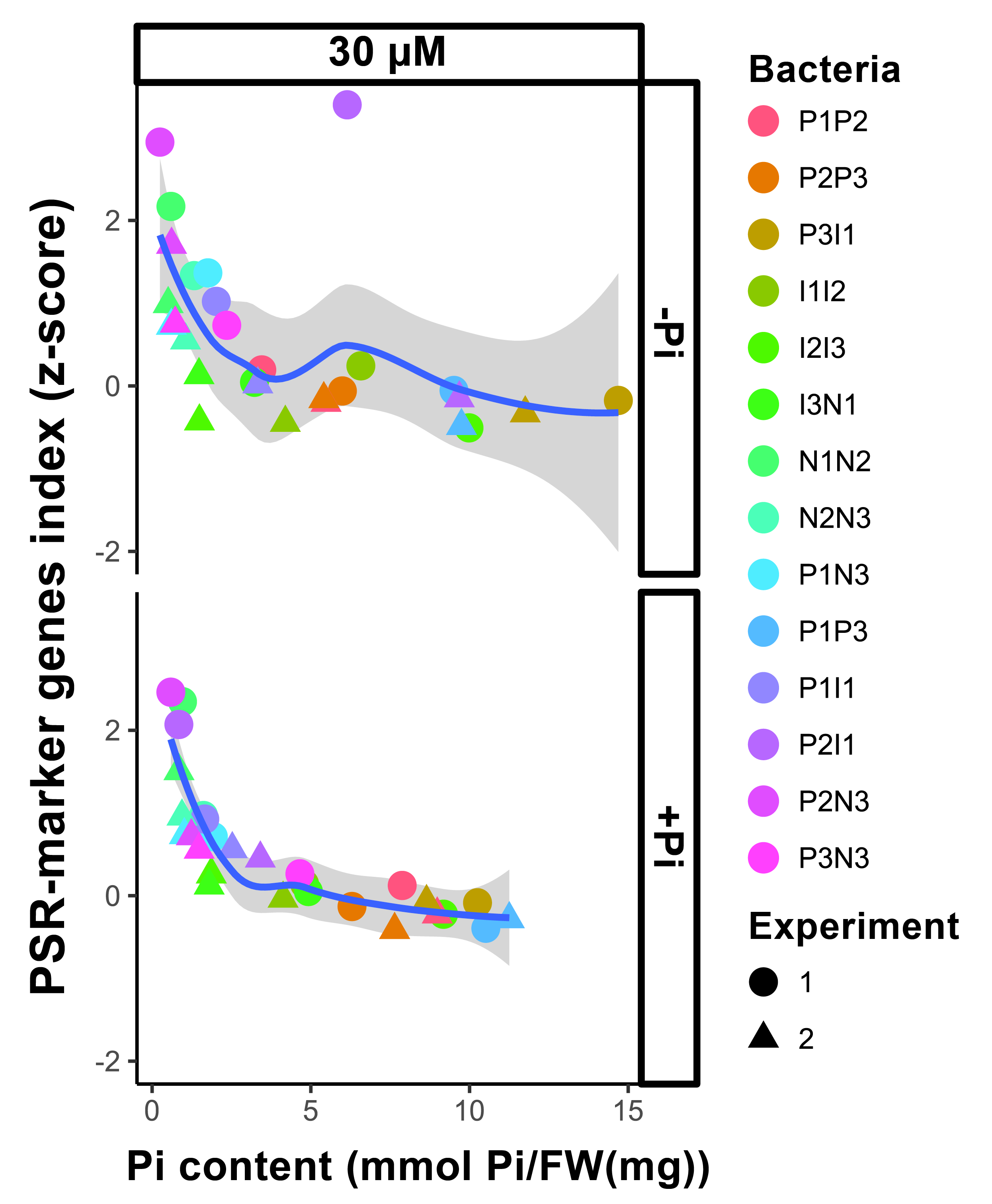

Supplement: S8 Fig — In the figure, each point represents a bacterial synthetic community. The x-axis gives the average of shoot Pi content and the y-axis the average of the phosphate starvation response marker genes activation. Data from 2 independent biological replicates are shown, and a loess smoother is shown as a blue line. The grey zone represents the 95% confidence interval of the smoother. Each panel represents a condition. Only conditions that ended with 30 μM Pi are shown, because no activation was observed in the other conditions. loess, local regression; Pi, phosphate. (TIF) [file pbio.2003962.s008.tif]

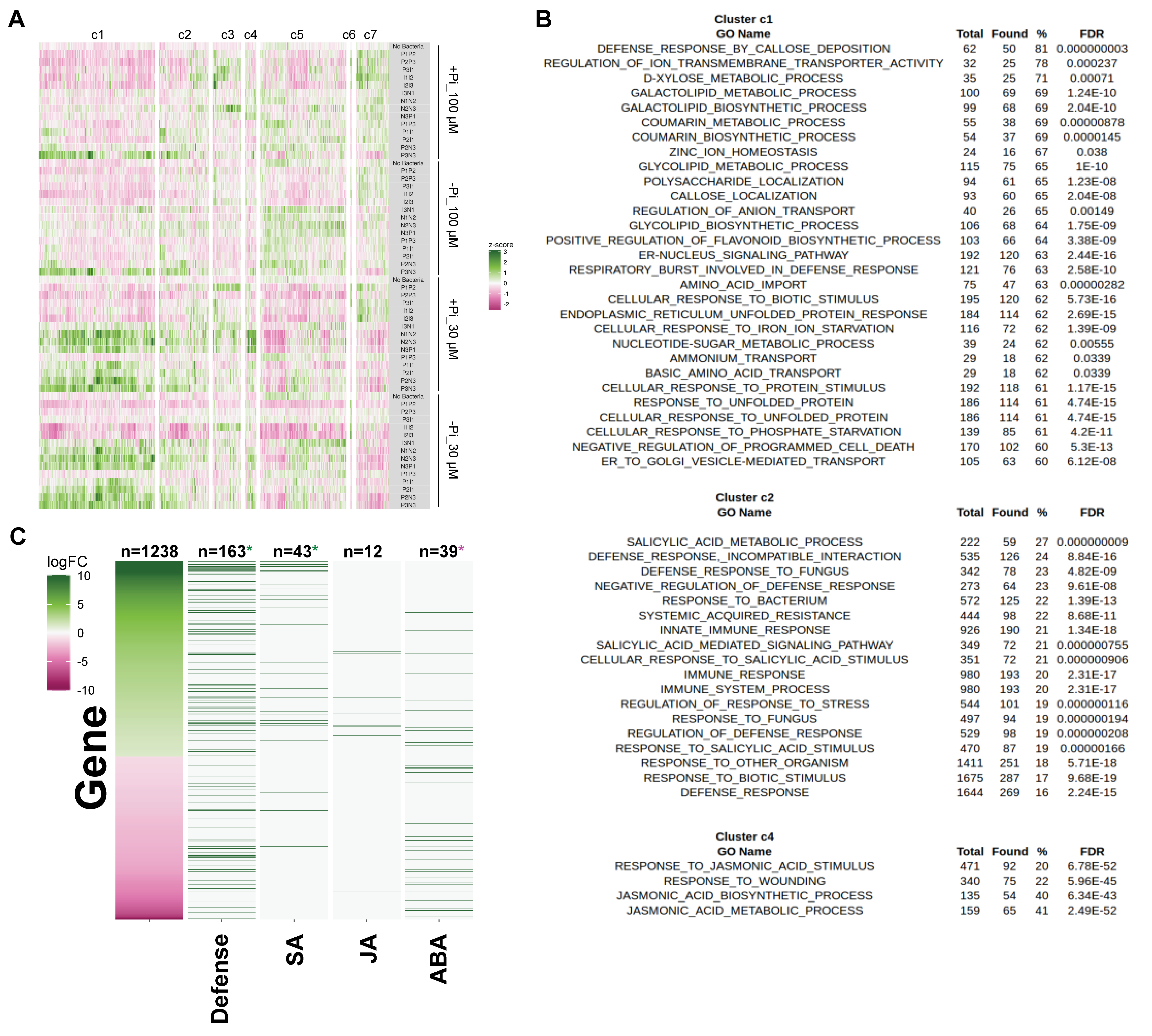

Supplement: S9 Fig — (A) Hierarchical clustering of the approximately 17,000 most variably expressed genes in our RNA-Seq experiment (S6 Table). Rows represent the average from all samples with a given bacterial treatment in each condition, and columns represent genes. Genes are clustered according to their expression profiles. (B) Gene ontology enrichments for clusters c1, c2, and c4 defined in (A). Gene sets (c1, c2) matched the expression pattern of the phosphate starvation response marker genes. Gene ontology enrichment analysis revealed that these clusters are enriched in defense genes (S7 Table). Cluster c1 also includes stress response genes, like low Pi responsive genes (S7 Table). Cluster c2 showed an overrepresentation of salicylic acid signaling genes and other genes associated with plant immunity (S7 Table). Cluster c3 contained numerous genes related to the metabolism of membrane phospholipids. These genes are more induced in 30 μM Pi and are potentially involved in the metabolic replacement of phospholipids by sulfolipids in Pi-deficient plants (S7 Table). We found in cluster c4 enrichment in plant immune system function, specifically in JA response. (C) Differentially expressed genes in response to bacteria are mainly associated with PTI and SA (S7 Table). The first column shows the log(fold-change) in the expression of 1,238 genes that are differentially expressed between plants that encountered bacteria versus axenically grown plants (FDR < 0.01). Positive values (green) correspond to genes more expressed in plants that encountered bacteria, while negative values (magenta) correspond to genes more highly expressed in axenically grown plants. The following columns indicate genes annotated as related to defense, SA, JA, or ABA, according to gene ontologies. The numbers on top indicate the number of genes in each functional class, and the asterisks indicate functions that are enriched among more highly expressed genes in the presence of bacteria (green) or in axenically gr [file pbio.2003962.s009.tif]

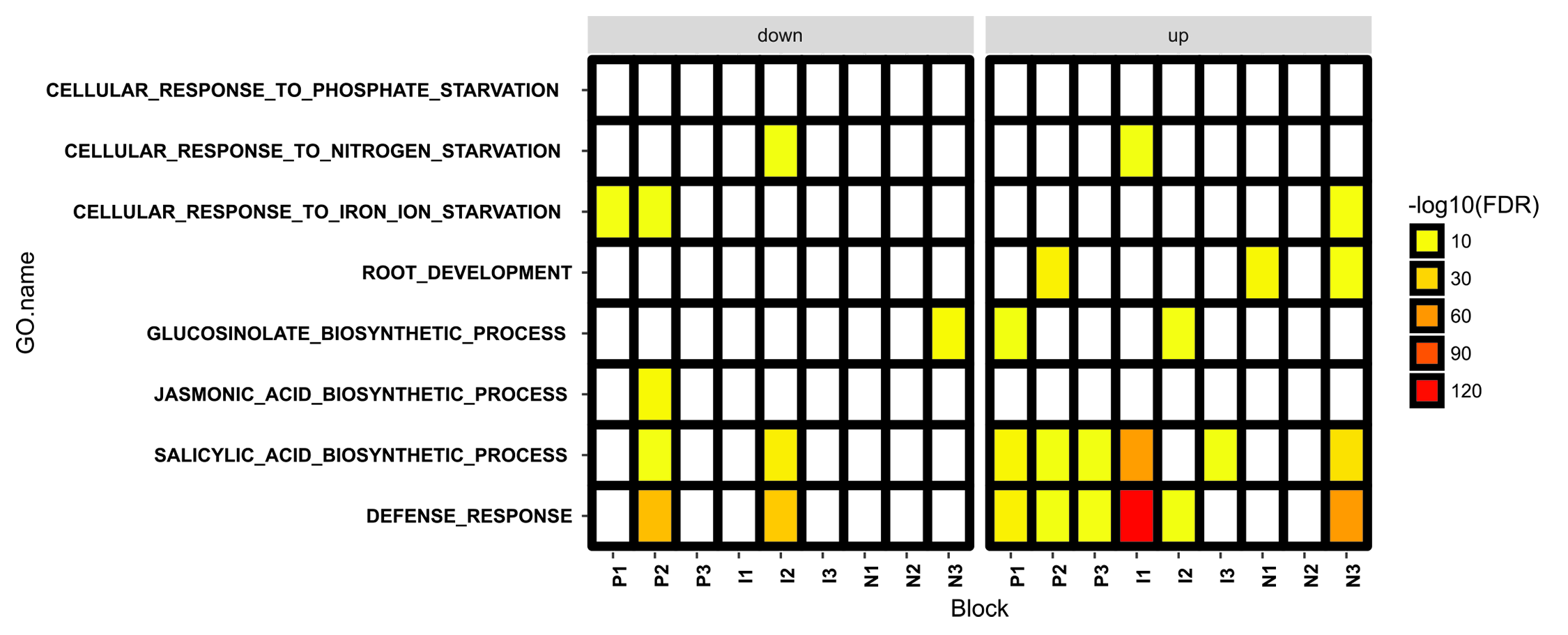

Supplement: S10 Fig — The left panel shows GO terms (GO names, y-axis) enrichments among down-regulated genes (down), and the right panel GO terms enrichments among up-regulated genes (up). The x-axis indicates the bacterial block for the corresponding enrichment test. Color indicates the statistical significance after controlling for multiple testing (−log10(FDR)). Nonsignificant enrichments (FDR ≥ 0.05) are shown in white. A significant result means that a particular GO term is enriched among the genes that are down-or up-regulated in response to specific bacterial blocks. Enrichment analysis was performed on differentially expressed genes from S6 Table. FDR, false discovery rate; GO, gene ontology. (TIF) [file pbio.2003962.s010.tif]

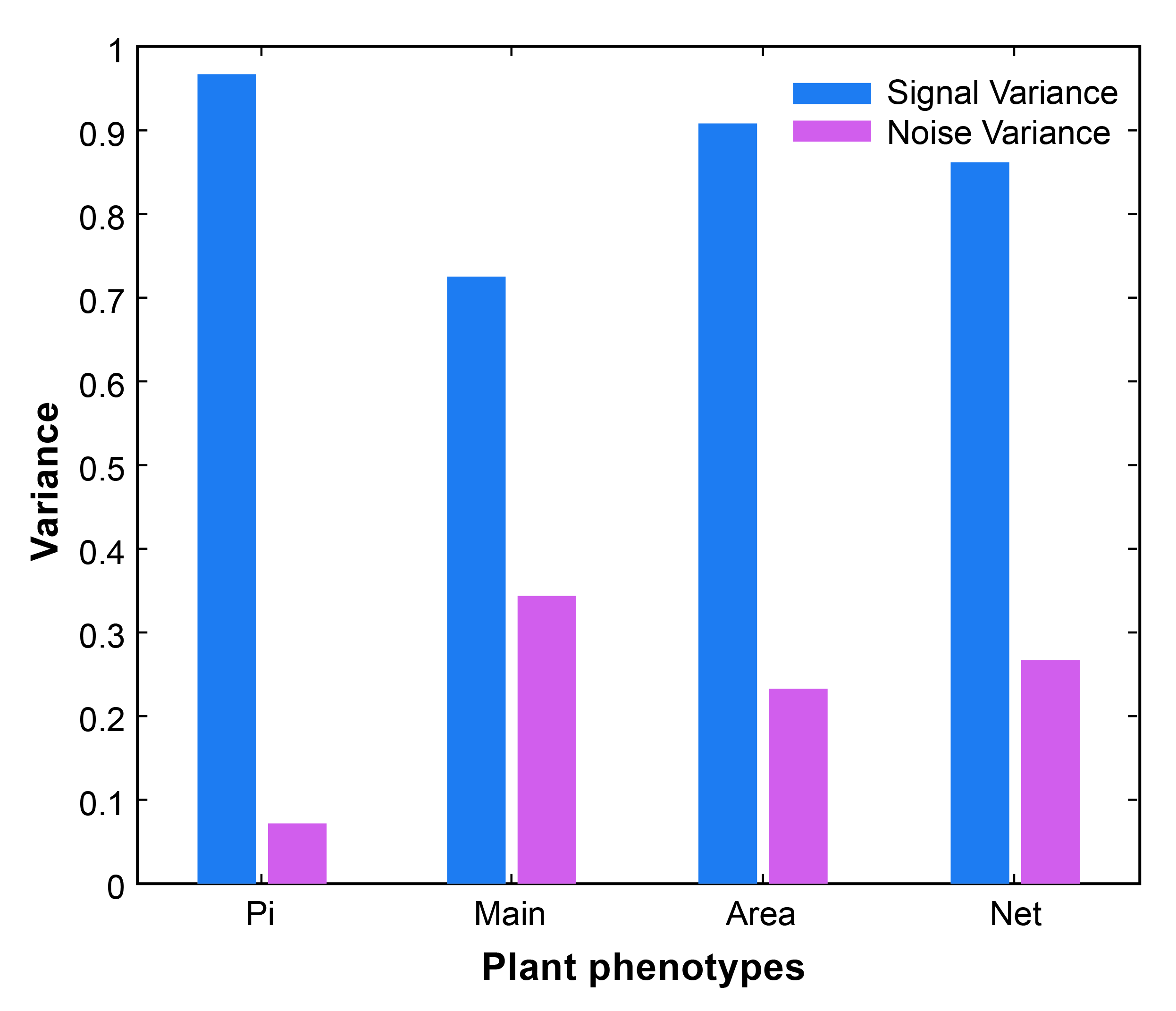

Supplement: S11 Fig — The bar plot shows the signal variance and noise variance for all plant phenotypes tested: shoot Pi content (Pi), Primary root elongation (Main), shoot area (Area), and total root network (Net). See also Materials and methods 4b. Numerical values that underlie the data displayed in the panel are in https://github.com/surh/wheelP. Pi, phosphate. (TIF) [file pbio.2003962.s011.tif]

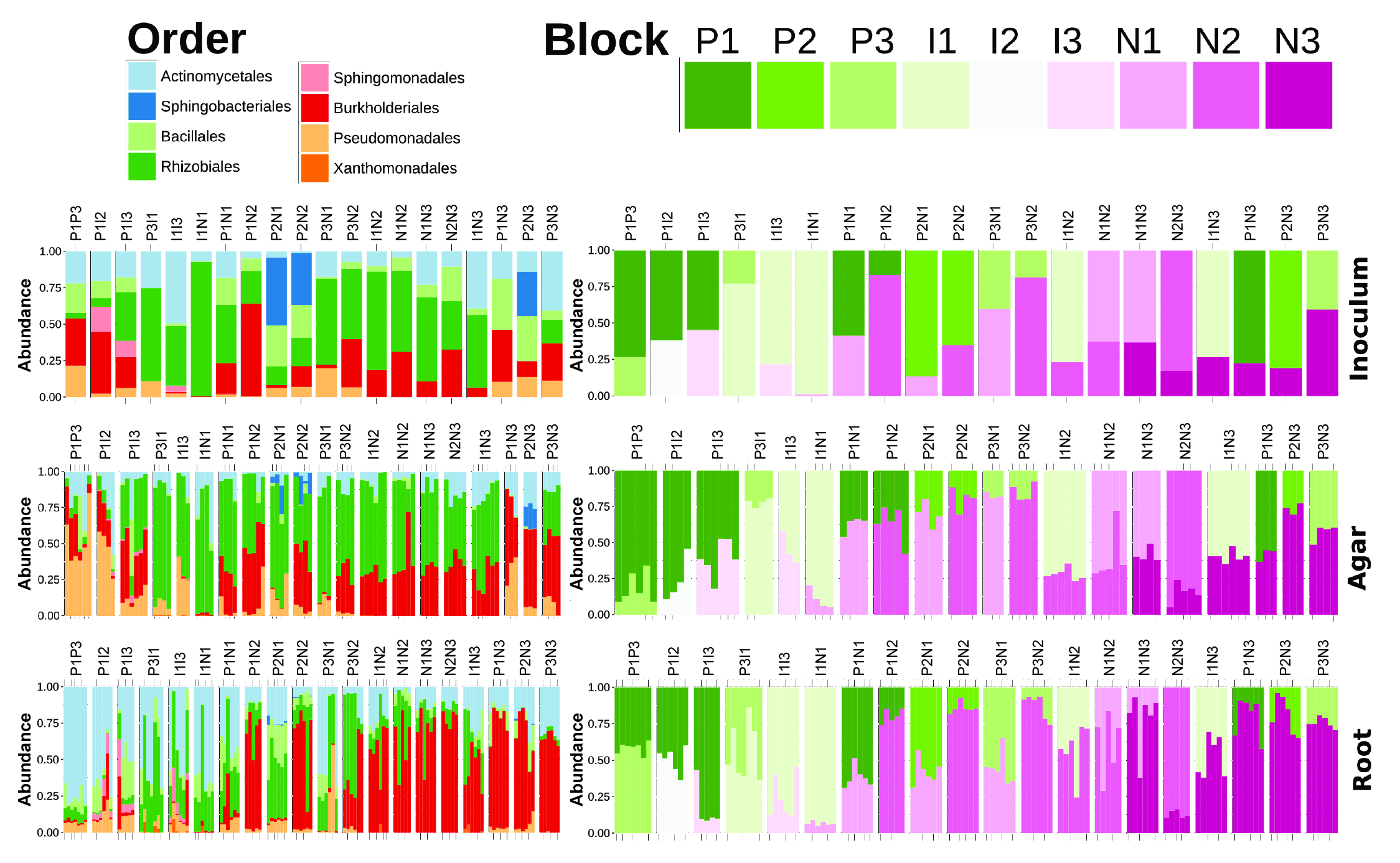

Supplement: S12 Fig — Taxonomic (left) and block (right) bacterial abundances for inoculum (top), agar (middle), and root (bottom) samples from the 20 synthetic communities of the validation experiment (see S4 Table for strains in each block). For each synthetic community, all individual sequenced samples with at least 400 reads are shown. For some synthetic communities, there were no samples that passed the minimum read threshold, and they are not shown. Colors indicate the proportion of bacterial reads that were mapped to the corresponding bacterial order (left) or block (right). All plants in this experiment were axenically germinated in a medium without phosphate supplementation (−Pi) and then transferred to 30 μM Pi, concomitant with addition of the different bacterial synthetic communities. Numerical values that underlie the data displayed in the panels are in https://github.com/surh/wheelP. Pi, phosphate. (TIF) [file pbio.2003962.s012.tif]
